# Lactate promotes endothelial-to-mesenchymal transition via Snail1 lactylation after myocardial infarction

**DOI:** 10.1126/sciadv.adc9465

**Published:** 2023-02-03

**Authors:** Min Fan, Kun Yang, Xiaohui Wang, Linjian Chen, P. Spencer Gill, Tuanzhu Ha, Li Liu, Nicole H. Lewis, David L. Williams, Chuanfu Li

**Affiliations:** ^1^ Department of Surgery, James H. Quillen College of Medicine, East Tennessee State University, Johnson City, TN 37614, USA.; ^2^ The Center of Excellence in Inflammation, Infectious Disease and Immunity, James H. Quillen College of Medicine, East Tennessee State University, Johnson City, TN 37614, USA.; ^3^ Xiamen Cardiovascular Hospital, Xiamen University, Xiamen, China.; ^4^ Department of Geriatrics, The First Affiliated Hospital of Nanjing Medical University, Nanjing, China.; ^5^ Department of Medical Education, James H. Quillen College of Medicine, East Tennessee State University, Johnson City, TN 37614, USA.

## Abstract

High levels of lactate are positively associated with the prognosis and mortality in patients with heart attack. Endothelial-to-mesenchymal transition (EndoMT) plays an important role in cardiac fibrosis. Here, we report that lactate exerts a previously unknown function that increases cardiac fibrosis and exacerbates cardiac dysfunction by promoting EndoMT following myocardial infarction (MI). Treatment of endothelial cells with lactate disrupts endothelial cell function and induces mesenchymal-like function following hypoxia by activating the TGF-β/Smad2 pathway. Mechanistically, lactate induces an association between CBP/p300 and Snail1, leading to lactylation of Snail1, a TGF-β transcription factor, through lactate transporter monocarboxylate transporter (MCT)–dependent signaling. Inhibiting Snail1 diminishes lactate-induced EndoMT and TGF-β/Smad2 activation after hypoxia/MI. The MCT inhibitor CHC mitigates lactate-induced EndoMT and Snail1 lactylation. Silence of MCT1 compromises lactate-promoted cardiac dysfunction and EndoMT after MI. We conclude that lactate acts as an important molecule that up-regulates cardiac EndoMT after MI via induction of Snail1 lactylation.

## Teaser

Lactate promotes fibrosis in heart attacks.

## INTRODUCTION

Myocardial infarction (MI) is a familiar manifestation induced by continuous ischemia of the heart or coronary arteries and is associated with high mortality (*1*, *2*). Cardiac fibrosis, a process of pathological extracellular matrix remodeling and activation of fibroblasts, is an unavoidable event in most cardiac injuries including MI (*3*–*5*). Although initially cardiac fibrosis prevents the infarcted heart tissues from rupturing during MI, continuous formation of cardiac fibrosis is almost always accompanied with a poor prognosis and eventually leads to the development of heart failure (*3*). Lactate is a ubiquitous intermediate of glycolysis, which was previously considered as a by-product of metabolism. However, growing evidence has revealed that lactate is involved in diverse disease processes such as tumor progression and sepsis (*6*–*10*). Clinical studies have shown that high lactate levels correlate with increased mortality in heart failure patients (*11*, *12*). In addition, lactic acid has been shown to induce myofibroblast differentiation and contributes to pulmonary fibrosis (*13*). Zhang *et al*. (*14*) revealed that glycolysis-derived lactate directly alters histones by adding lactyl group to histone lysine (K) residues, a process that is termed as “lactylation,” which ultimately affects gene transcription. It is well known that epigenetic modification controls profibrotic gene expression during cardiac fibrosis (*15*). However, whether lactate will induce lactylation and cardiac fibrosis after myocardial ischemic injury remains unknown.

Endothelial-to-mesenchymal transition (EndoMT) is a process that endothelial cells undergo in which a series of cellular and molecular changes lead to an alteration in phenotype toward mesenchymal cells, such as myofibroblasts (*16*). Cardiac hypoxia following MI promotes EndoMT, which contributes to cardiac fibrosis, thus leading to cardiac dysfunction (*17*, *18*). Previous investigations show evidence that EndoMT contributes to cardiac fibrosis after MI and inhibition of EndoMT attenuates cardiac fibrosis (*17*, *19*, *20*). Transforming growth factor–β (TGF-β) has been widely reported to play a critical role in EndoMT-related fibrosis in various cardiovascular diseases (*19*, *21*). Activation of TGF-β signaling stimulates the phosphorylation of Smad2/3, as well as the expression of EndoMT-promoting transcription factors, such as Snail1 (*22*). Snail1 can govern EndoMT during endocardial cushion formation in the heart (*23*).

Here, we observed that increased lactate levels strongly enhanced cardiac fibrosis and exacerbated cardiac dysfunction by inducing EndoMT in the myocardium following MI. In vitro, we found that lactate promotes hypoxia-induced EndoMT and TGF-β/Smad2 signaling activation in endothelial cells. Mechanistically, we demonstrated that lactate up-regulated Snail1 nuclear translocation and lactylation after hypoxia/MI. Inhibition of Snail1 improved lactate-induced cardiac dysfunction following MI, suppressed lactate-stimulated EndoMT, lactylation of Snail1, and activation of TGF-β/Smad2 signaling. These findings enhance our understanding of the role of lactate in EndoMT following MI and will be the basis for the development of innovative therapies for improving cardiac remodeling and function after MI.

## RESULTS

### Reduction of lactate improved cardiac function and cardiac fibrosis following MI

**Fig. 1.**
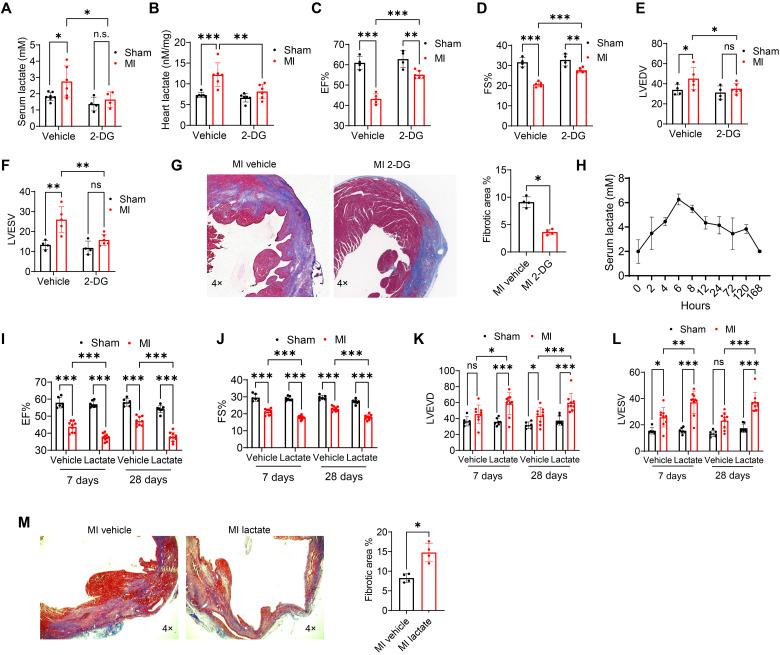
Increased lactate levels result in worsened cardiac dysfunction and increased cardiac fibrosis after MI. Intraperitoneal injection of 2-DG or vehicle was administrated to wild-type mice daily since 1 day before MI or sham surgery. Three days after surgery, serum (**A**) and cardiac (**B**) lactate levels were measured by commercially available kit (*n* = 4 to 7 per group). (**C** to **F**) Left ventricular (LV) ejection fraction (EF%), fractional shortening (FS%), LV end-diastolic volume (LVEDV), and LV end-systolic volume (LVESV) were tested 7 days after MI or sham surgical operation (*n* = 4 to 6 per group). (**G**) To examine cardiac fibrosis, cardiac sections were stained with trichrome stain (Masson) kit (*n* = 4 per group). In separate experiments, mice were subjected to MI or sham surgery followed by supplemental lactate administration (intraperitoneal injection) every 7 days for 28 days. (**H**) Serum lactate levels were examined at 2, 4, 6, 8, 12, 24, 72, 120, and 168 hours after lactate injection (*n* = 3 per group). (**I** to **L**) To evaluate cardiac function, EF%, FS%, LVEDV, and LVESV were tested 7 and 28 days after MI or sham surgical operation (*n* = 6 to 10 per group). (**M**) Cardiac sections were stained with trichrome stain (Masson) kit to test cardiac fibrosis (*n* = 4 per group). Comparisons of data between groups were made using two-way analysis of variance (ANOVA) followed by Tukey’s procedure. *P < 0.05, **P < 0.01, ***P < 0.001 compared with indicated groups. ns, not significant.

Clinical studies have revealed that high lactate levels are prevalent in heart failure patients and are correlated with the mortality of heart failure (*11*, *12*). In the present study, we examined whether MI could increase circulating lactate levels. As shown in Fig. 1 (A to F), MI significantly promoted both serum and cardiac lactate levels accompanied with decreased cardiac function 7 days after surgery. To determine whether reduction of lactate would ameliorate cardiac dysfunction and the process of fibrosis in the myocardium after MI, the glycolysis inhibitor 2-deoxy-d-glucose (2-DG) (*24*) was administrated by intraperitoneal injection to decrease lactate levels. 2-DG suppressed serum and cardiac lactate levels induced by MI (Fig. 1, A and B). Of interest, the ejection fraction (EF%) and fractional shortening (FS%) levels were higher in 2-DG MI mice than in vehicle MI mice (Fig. 1, C and D). The left ventricular end-diastolic volume (LVEDV) and LV end-systolic volume (LVESV) values were lower in 2-DG MI mice than in vehicle MI mice (Fig. 1, E and F), indicating that suppressed lactate production improves cardiac function after MI. In addition, Masson’s trichrome staining showed that 2-DG administration resulted in less cardiac fibrosis after MI (Fig. 1G). These data indicate that increased lactate production may contribute to MI-induced fibrosis and cardiac dysfunction.

### Increased lactate production worsened cardiac dysfunction and increased cardiac fibrosis after MI

To confirm the role of lactate in cardiac dysfunction and fibrosis after MI, we administrated supplemental lactate to mice 3 hours after induction of MI and assessed cardiac function with echocardiography 1 and 4 weeks after MI. Figure 1H shows that serum lactate concentration returned to normal levels 7 days after intraperitoneal injection of lactate (0.5 g/kg body weight). Therefore, lactate was administered by intraperitoneal injection every 7 days. Compared with the sham group, MI markedly down-regulated the values of EF% and FS% (Fig. 1, I and J). We observed that administration of lactate further depressed EF% (by 14.0% at 7 days and 19.5% at 28 days) and FS% (by 15.8% at 7 days and 21.6% at 28 days) values when compared with the MI group (Fig. 1, I and J). In parallel, treatment with lactate increased the levels of LVEDV and LVESV after MI (Fig. 1, K and L). We also used an osmotic mini-pump to consistently maintain stable serum lactate levels in mice. As shown in fig. S1A, lactate administration by mini-pump promoted serum lactate levels both under normal conditions and after MI. As expected, elevated lactate levels markedly down-regulated the levels of EF% and FS% (fig. S1, B and C) and up-regulated values of LVEDV and LVESV (fig. S1, D and E) following MI. In addition, treatment of 2-DG attenuated MI-induced cardiac infarct size, while supplemental lactate administration further promoted cardiac infarct size after MI (fig. S2A). Figure S3A showed that lactate enhanced terminal deoxynucleotidyl transferase–mediated deoxyuridine triphosphate nick end labeling (TUNEL)–positive staining in the myocardium after MI. On the other hand, 2-DG administration decreased TUNEL-positive staining in MI-treated heart (fig. S3A), indicating that increased lactate levels may increase cardiac apoptosis after MI. Together, these data indicate that increasing lactate levels worsens cardiac dysfunction with following MI. Masson’s trichrome staining provided evidence that lactate administration resulted in a significant induction of cardiac fibrosis in MI-treated mice (Fig. 1M).

### Suppression of lactate production attenuated cardiac EndoMT after MI

**Fig. 2.**
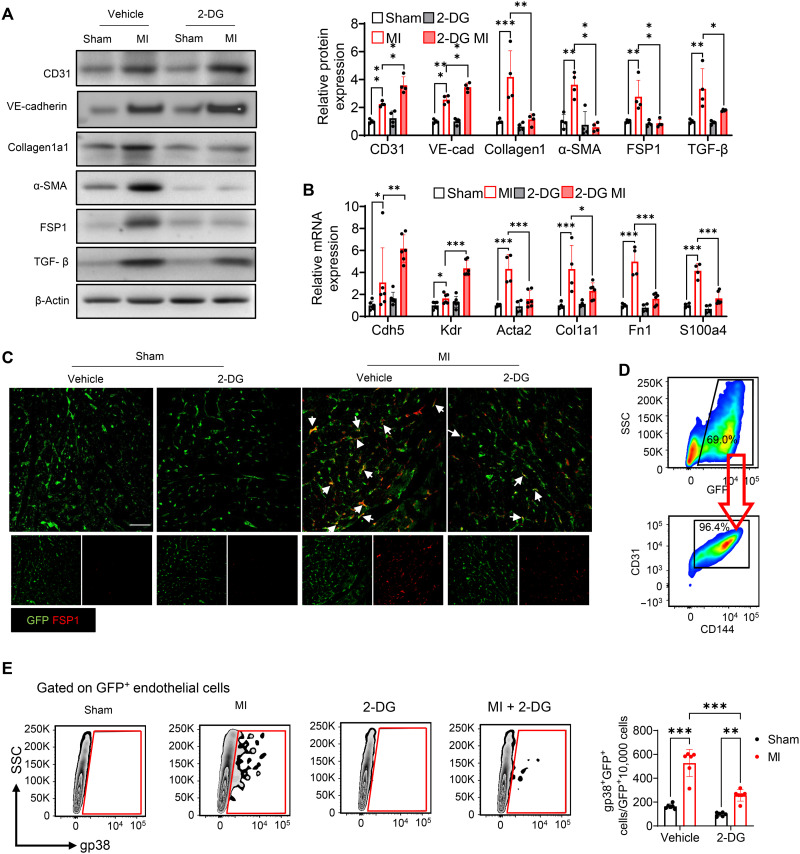
2-DG attenuates MI-induced EndoMT. 2-DG or vehicle was administrated to wild-type mice via intraperitoneal injection daily since 1 day before MI or sham surgery, and heart tissues were harvested. (**A**) The expression of endothelial cell marker CD31, VE-cadherin, mesenchymal marker Collagen1a1, α-SMA, FSP1, and TGF-β in the myocardium was measured by Western blot (*n* = 4 per group). (**B**) Expression analysis by qRT-PCR of endothelial marker *Cdh5* and *Kdr* and mesenchymal marker *Acta2*, *Col1a1*, *Fn1*, and *S100a4* mRNA from the myocardium of MI or sham mice with 2-DG or vehicle administration (*n* = 4 to 6 per group). Endothelial cell–specific GFP-labeled (TIE2GFP) mice were subjected to MI or sham surgery. (**C**) Representative immunofluorescent staining images of GFP-labeled endothelial cell (green) and fibroblast marker FSP1 (red) in the heart tissues of TIE2GFP mice (*n* = 4 per group). Scale bar, 50 μm. (**D**) GFP-positive endothelial cells from heart tissues of TIE2GFP mice were also positive for endothelial cell marker anti-CD31 and anti-CD144 antibodies. (**E**) Representative flow density plot and quantitative analysis for gp38-positive endothelial cell frequency in all GFP-positive endothelial cells from heart tissues of TIE2GFP mice. *n* = 4 per group. Comparisons of data between groups were made using two-way ANOVA followed by Tukey’s procedure. *P < 0.05, **P < 0.01, ***P < 0.001 compared with indicated groups.

EndoMT, a process through which endothelial cells lose their endothelial characteristics and transit to a fibroblast-like phenotype, plays a critical role in cardiac fibrosis after MI (*25*). To investigate how lactate increased cardiac fibrosis after MI, we next evaluated whether there is an association between lactate and cardiac EndoMT after MI. MI induced significant increases in both endothelial marker CD31 and VE-cadherin expression (Fig. 2A and fig. S4, A and B) and mesenchymal marker fibroblast-specific protein 1 (FSP1), α-smooth muscle actin (α-SMA), and Collagen1a1 expression (Fig. 2A), when compared with sham control. However, suppression of lactate production by administration of 2-DG elevated the expression of CD31 and VE-cadherin while eliminating the increase of Collagen1a1, α-SMA, and FSP1 induced by MI (Fig. 2A). In accordance, treatment with 2-DG up-regulated mRNA levels of endothelial markers *Cdh5* and *Kdr* (Fig. 2B). On the contrary, 2-DG down-regulated mesenchymal marker *Atca2*, *Col1a1*, *Fn1*, and *S100a4* mRNA expression (Fig. 2B). Together, these data suggest that inhibition of lactate attenuates the process of EndoMT following MI.

To confirm our observation, we induced MI in endothelial cell–specific green fluorescent protein (GFP)–labeled mice (TIE2GFP) supplemented with or without 2-DG and performed immunofluorescent staining in the heart tissues with anti-GFP (green) and anti-FSP1 (red) antibodies. As shown in Fig. 2C, MI induced colocalization of GFP with FSP1 when compared with the sham control group. In contrast, inhibition of lactate production by 2-DG attenuated MI-induced colocalization between GFP and FSP1, indicating that 2-DG decreases MI-stimulated EndoMT in the myocardium. In addition, 7 days after MI or sham surgery, we isolated endothelial cells from the harvested hearts and examined the percentage of endothelial cells differentiating into myofibroblasts by flow cytometry. As shown in Fig. 2D, endothelial cells isolated from sham control mice show that the most GFP cells were stained with the anti-CD31 and anti-CD114 (endothelial cell markers) antibodies. Gp38 is a well-accepted marker for fibroblast populations in heart (*26*). Figure 2E shows that there was almost no detectable Gp38 staining in endothelial cells isolated from sham control hearts. However, MI markedly promoted numbers and percentage of Gp38-positive staining cells in the GFP-labeled endothelial cells. On the contrary, treatment with 2-DG suppressed MI-induced Gp38-positive endothelial cells.

### High levels of lactate promote EndoMT in the myocardium following MI

**Fig. 3.**
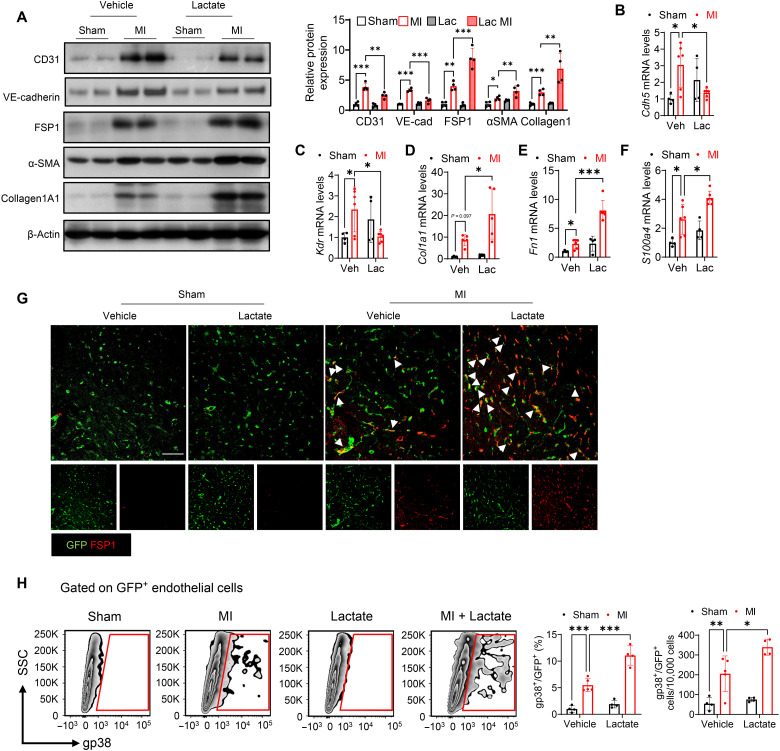
Supplemental lactate promotes EndoMT following MI. Mice were subjected to MI or sham surgery followed by supplemental lactate administration (intraperitoneal injection, 0.5 g/kg body weight). (**A**) The expression of endothelial cell markers CD31 and VE-cadherin and mesenchymal markers FSP1, α-SMA, and Collagen1a1 in the myocardium was measured by Western blot (*n* = 4 per group). (**B** to **F**) Expression analysis by qRT-PCR of endothelial marker *Cdh5* and *Kdr* and mesenchymal marker *Col1a1*, *Fn1*, and *S100a4* mRNA from the myocardium of MI or sham mice with lactate or vehicle administration (*n* = 4 to 6 per group). TIE2GFP mice were subjected to MI or sham surgery followed by lactate or vehicle administration. (**G**) Representative immunofluorescent staining images of GFP-labeled endothelial cell (green) and fibroblast marker FSP1 (red) in the heart tissues of TIE2GFP mice. Scale bar, 50 μm. (**H**) Representative flow density plot and quantitative analysis for gp38-positive endothelial cell frequency in all GFP-positive endothelial cells from heart tissues of TIE2GFP mice. *n* = 4 to 5 per group. Comparisons of data between groups were made using two-way ANOVA followed by Tukey’s procedure. *P < 0.05, **P < 0.01, ***P < 0.001 compared with indicated groups.

We then examined whether supplemental lactate production could promote the EndoMT transition following MI. Western blot showed that supplemental lactate to MI mice reduced the levels of endothelial cell markers (CD31 and VE-cadherin) and promoted the expression of mesenchymal markers (FSP1, α-SMA, and Collagen1a1) (Fig. 3A), when compared with the MI group. Consistently, quantitative real-time polymerase chain reaction (qRT-PCR) showed that enhanced lactate levels led to a reduction of *Cdh5* and *Kdr* mRNA levels (Fig. 3, B and C) and an induction of *Col1a1*, *Fn1*, and *S100a4* mRNA levels (Fig. 3, D to F) after MI, suggesting that lactate induces EndoMT after MI.

Next, TIE2GFP mice were subjected to MI or sham surgery followed by lactate administration. Immunofluorescent staining with anti-GFP (green) and anti-FSP1 (red) antibodies showed that MI induced colocalization of GFP and FSP1 when compared with sham mice (Fig. 3G). Of significance, lactate treatment resulted in markedly more colocalization of GFP and FSP1 after MI (Fig. 3G), showing that there are more endothelial cells differentiated into fibroblasts with lactate administration. Similarly, flow cytometry showed that treatment with lactate further up-regulated the population of GFP^+^gp38^+^ cells after MI (Fig. 3H), indicating that lactate promoted EndoMT following MI.

To further elucidate the role of lactate in EndoMT after MI, we isolated endothelial cells from heart tissues of mice subjected to MI or sham surgery. As shown in fig. S5A, MI promoted Collagen1a1, α-SMA, and FSP1 expression in cardiac endothelial cells. Treatment of lactate further stimulated the expression of these mesenchymal markers after MI. In contrast, 2-DG strongly blocked Collagen1a1, α-SMA, and FSP1 expression induced by MI. Consistently, the mRNA levels of *Acta2*, *Col1a1*, *Fn1*, and *S100a4* were significantly increased with administration of lactate and decreased by 2-DG administration after MI (fig. S5, B to E).

### Lactate stimulated endothelial cell migration, decreased VE-cadherin expression, and promoted EndoMT after hypoxia

**Fig. 4.**
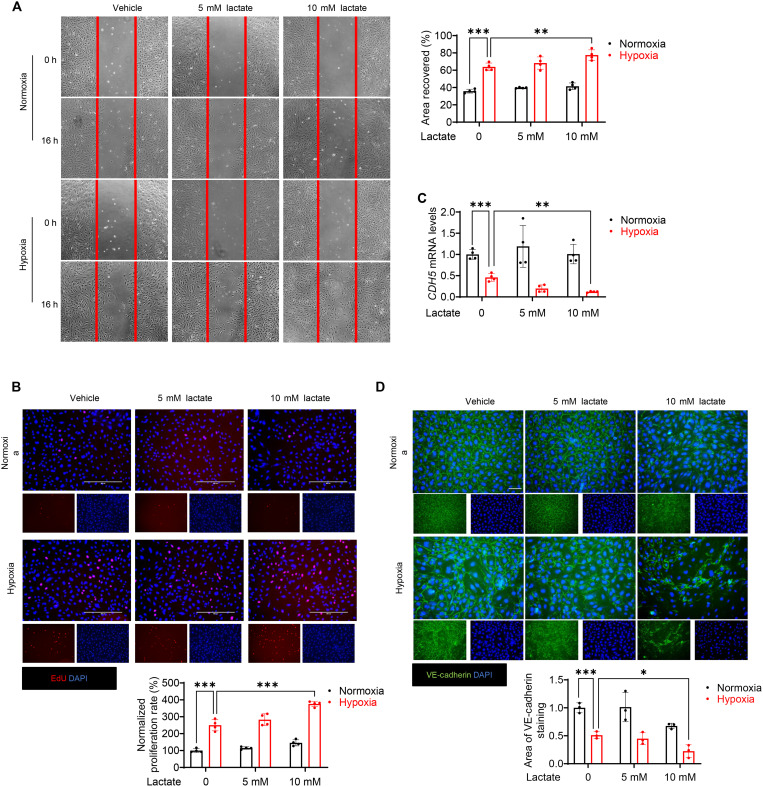
Lactate induces endothelial cell migration and decreases VE-cadherin expression after hypoxia. HUVECs were treated with lactate (5 or 10 mM) followed by normoxic or hypoxic challenge. (**A**) Endothelial cell migration was measured by wound-healing (or scratch) assay. Original magnification, ×20. (**B**) Endothelial cell proliferation was measured by EdU staining. Expression analysis by qRT-PCR (**C**) and immunofluorescent staining (**D**) of VE-cadherin (green) and nuclei (DAPI, blue). Scale bars, 50 μm. *n* = 3 per group. Comparisons of data between groups were made using one-way ANOVA followed by Tukey’s procedure. *P < 0.05, **P < 0.01, ***P < 0.001 compared with indicated groups.

To define the mechanisms by which lactate promotes the EndoMT process, we perfomed in vitro experiments using endothelial cells subjected to hypoxic challenge. It is well known that endothelial cell migration and proliferation are associated with the process of EndoMT (*25*, *27*, *28*). Therefore, we performed wound-healing assay and 5-ethynyl-2-deoxyuridine (EdU) incorporation assay to see whether lactate would alter endothelial cell migration and proliferation. Figure 4A shows that hypoxia accelerated endothelial cell migration when compared with the normoxia control. Furthermore, administration of lactate at 10 mM, but not 5 mM, significantly enhanced wound closure induced by hypoxia (Fig. 4A), indicating that lactate induces endothelial cell migration following hypoxic challenge. Similarly, EdU incorporation assay revealed that lactate can promote hypoxia-stimulated endothelial cell proliferation (Fig. 4B).

**Fig. 5.**
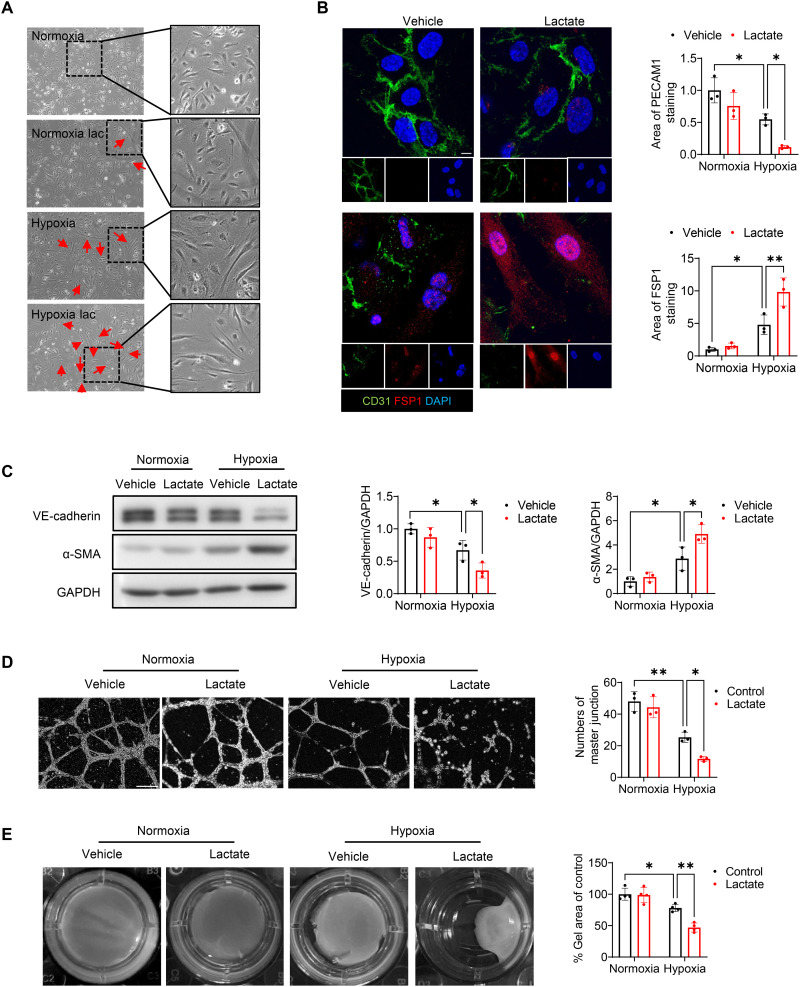
Lactate promotes EndoMT in endothelial cells after hypoxia. HUVECs were treated with lactate (10 mM) followed by normoxic or hypoxic challenge. (**A**) Morphology of endothelial cells. Original magnification, ×20. *n* = 3 per group. (**B**) Immunofluorescent staining of CD31 (green), FSP1 (red), and nuclei (DAPI, blue). Scale bar, 10 μm. *n* = 3 per group. (**C**) The expression of endothelial marker VE-cadherin and mesenchymal marker α-SMA in endothelial cells was measured by Western blot (*n* = 3 per group). (**D**) Endothelial cell angiogenesis was examined by Matrigel assay (*n* = 3 per group). Scale bar, 200 μm. (**E**) Collagen gel contraction assay was performed to test fibroblast function (*n* = 4 per group). Comparisons of data between groups were made using two-way ANOVA followed by Tukey’s procedure. *P < 0.05, **P < 0.01 compared with indicated groups.

Next, we examined the levels of VE-cadherin in the migrated cells to disclose whether lactate could alter endothelial cell phenotype after stimulation of their migration following hypoxia. Immunofluorescent staining of VE-cadherin showed that the integrity of VE-cadherin was dampened 72 hours after hypoxia (Fig. 4D). Treatment with 10 mM lactate further disengaged VE-cadherin integrity on the endothelial cell surface after hypoxia (Fig. 4D). In addition, Western blot and qRT-PCR analysis also showed that lactate treatment markedly decreased the levels of VE-cadherin, when compared with the hypoxia group (Figs. 4C and 5C), demonstrating that lactate may alter the phenotype of endothelial cells under hypoxic challenge.

To illustrate whether lactate could induce EndoMT in endothalial cells following hypoxia, we treated endothelial cells with lactate and examined the markers for EndoMT after hypoxia. As shown in Fig. 5A, the morphology of endothelial cells changed from the typical shape to an elongated, spindle appearance after hypoxia for 72 hours. Treatment with lactate resulted in greater changes of cell morphology than in hypoxic control. Furthermore, the elongated cells stained positively for FSP1 and negatively for CD31 (Fig. 5B). In accordance, Fig. 5C shows that administration of lactate reduced VE-cadherin expression and induced α-SMA expression after hypoxic challenge. To investigate whether lactate-promoted EndoMT would induce endothelial cell dysfunction, we performed a Matrigel-based endothelial cell angiogenesis assay and found that lactate administration led to the repression of tube formation induced by hypoxia (Fig. 5D). In addition, using the collagen gel contraction assay, we confirmed that lactate-treated endothelial cells exhibited a mesenchymal-like function following hypoxia stimulation, as evidenced by reduced collagen gel size and increased contractility (Fig. 5E). Consistently, we observed that hypoxia remarkably decreased the mRNA levels of *CDH5* and *KDR* (fig. S6, A and B) but increased *ATCA2*, *COL1A1*, *FN1*, and *S100A4* expression in human cardiac microvascular endothelial cells (HCMECs) (fig. S6, C to F). Treatment with lactate further down-regulated endothelial marker expression and up-regulated mesenchymal marker expression (fig. S6, A to F). Together, these data indicate that lactate induces EndoMT following hypoxia.

### Lactate activated TGF-β/Smad2 signaling after hypoxia

**Fig. 6.**
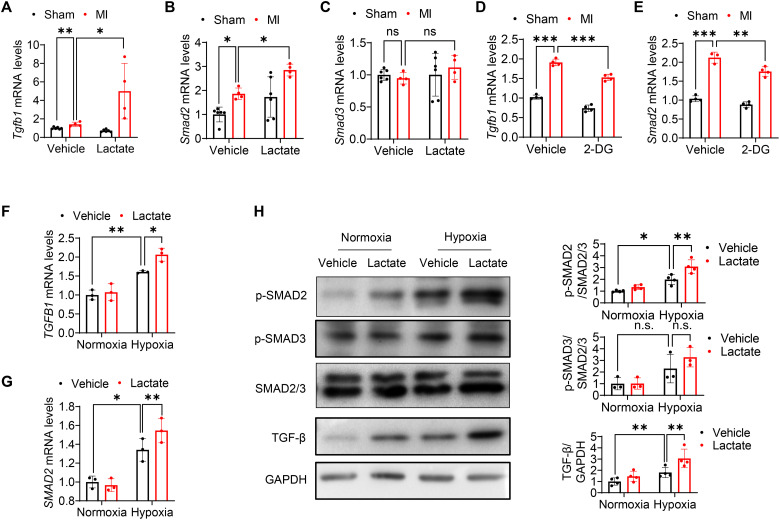
Lactate stimulates TGF-β/Smad2 signaling after MI/hypoxia. (**A** to **C**) Mice were subjected to MI or sham surgery followed by supplemental lactate administration. The mRNA expression of *Tgfb1*, *Smad2*, and *Smad3* was examined by qRT-PCR (*n* = 4 to 6 per group). (**D** and **E**) In separate experiments, mice were subjected to MI or sham surgery with 2-DG or vehicle administration. The mRNA expression of *Tgfb1* and *Smad2* was examined by qRT-PCR (*n* = 3 to 4 per group). (**F** to **H**) HUVECs were treated with lactate (10 mM) followed by normoxic or hypoxic challenge. The mRNA levels of *TGFB1* and *SMAD2* were examined by qRT-PCR. The expression of phospho (p)–SMAD2, phospho-SMAD3, and TGF-β was measured by Western blot (*n* = 3 to 4 per group). Comparisons of data between groups were made using two-way ANOVA followed by Tukey’s procedure. *P < 0.05, **P < 0.01, ***P < 0.001 compared with indicated groups.

To define the mechanisms by which lactate induces EndoMT following hypoxia, we evaluated TGF-β and Smad2/3 expression, which are known to be involved in regulating EndoMT (*19*, *25*). In vivo, qRT-PCR showed that lactate significantly accelerated *Tgfb1* and *Smad2* mRNA expression in the myocardium after MI (Fig. 6, A and B). However, it did not alter the mRNA levels of *Smad3* (Fig. 6C). Similar results were observed in endothelial cells isolated from the heart tissues (fig. S7, A to C). In addition, inhibition of lactate production by 2-DG mitigated MI-induced *Tgfb1* and *Smad2* mRNA expression (Fig. 6, D and E). In vitro study also revealed that lactate promoted *TGFB1* and *SMAD2* mRNA expression in both human umbilical cord endothelial cells (HUVECs) and HCMECs subjected to hypoxic challenge (Fig. 6, F and G, and fig. S6, G and H). Consistent with gene expression levels, treatment with lactate up-regulated phosphorylation of Smad2 and expression of TGF-β but did not affect the phosphorylation of Smad3 following hypoxic challenge (Fig. 6H). Combined, these data indicate that lactate-induced TGF-β/Smad2 activation may contribute to EndoMT following hypoxia.

### Inhibition of intracellular lactate attenuated hypoxia-induced EndoMT

**Fig. 7.**
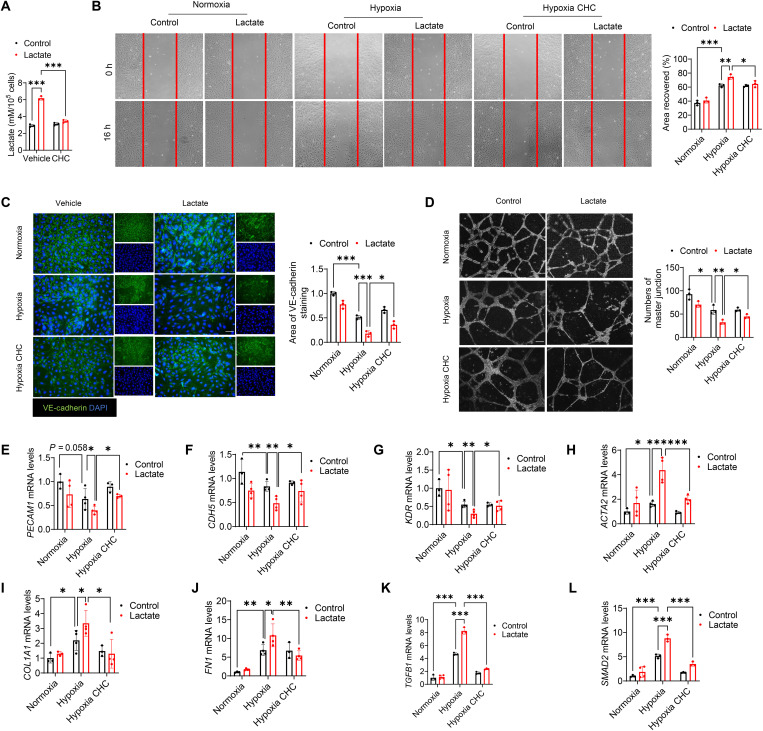
Inhibition of intracellular lactate attenuates hypoxia-induced EndoMT. Endothelial cells were treated with the lactate transporter MCT inhibitor α-cyano-4-hydroxycinnamate (CHC) before lactate administration. (**A**) Intracellular lactate levels were measured by commercially available kit. (**B**) Endothelial cell migration was measured by wound-healing assay. Original magnification, ×20. (**C**) Immunofluorescent staining of VE-cadherin (green) and nuclei (DAPI, blue). Scale bar, 50 μm. (**D**) Endothelial cell angiogenesis was examined by Matrigel assay. Scale bar, 100 μm. (**E** to **L**) The mRNA levels of *PECAM1*, *CDH5*, *KDR*, *ACTA2*, *FN1*, *COL1A1*, *TGFB1*, and *SMAD2* were examined by qRT-PCR. *n* = 3 to 4 per group. Comparisons of data between groups were made using two-way ANOVA followed by Tukey’s procedure. *P < 0.05, **P < 0.01, ***P < 0.001 compared with indicated groups.

Monocarboxylate transporters (MCTs) are bidirectional transporters that transport lactate across plasma membranes (*29*). By using the MCT inhibitor α-cyano-4-hydroxycinnamate (CHC) (*30*) before lactate administration, we observed that CHC abrogated intracellular lactate levels induced by lactate treatment (Fig. 7A), indicating that extracellular lactate is transported into endothelial cells via MCTs. In addition, CHC administration ameliorated lactate-promoted endothelial cell migration after hypoxia (Fig. 7B). Immunofluorescent staining showed that CHC prevented the reduction of VE-cadherin expression by lactate treatment (Fig. 7C), while tube formation assay indicated that CHC promoted angiogenesis after hypoxia (Fig. 7D). Moreover, CHC alleviated the decrease of endothelial marker *PECAM1*, *CDH5*, and *KDR* mRNA levels (Fig. 7, E to G) as well as the increase of mesenchymal marker *ACTA2*, *FN1*, and *COL1A1* mRNA levels (Fig. 7, H to J) induced by lactate. Accompanying these changes, treatment with CHC also repressed lactate-induced *TGFB1* and *SMAD2* mRNA expression (Fig. 7, K and L).

To confirm the role of MCT in lactate-stimulated EndoMT, endothelial cells were transfected with small interfering RNA (siRNA) specific for MCT1 to knock down the expression of MCT1 (fig. S8A), followed by lactate and hypoxic stimulation. Deficiency of MCT1 restored endothelial cell markers (*PECAM1* and *KDR*) suppressed by lactate administration (fig. S8, B and C). In contrast, silencing of MCT1 alleviated lactate-induced mesenchymal marker expression (*ACTA2*, *COL1A1*, *FN1*, and *COL1A1*) (fig. S8, D to G) as well as *TGFB1* and *SMAD2* mRNA levels (fig. S8, H and I).

Next, we performed in vivo transfection of siSlc16a1 by administration to the myocardium through the right common carotid artery before induction of MI. As shown in fig. S9 (A and B), administration of siSlc16a1 markedly decreased MCT1 expression in the myocardium. Seven days after surgery, cardiac function was measured by echocardiograph. As shown in fig. S10 (A to I), siSlc16a1 administration alone did not alter cardiac function and the process of EndoMT, when compared with sham control. However, silencing of MCT1 attenuated the down-regulation of EF% and FS% and inhibited the up-regulation of LVEDV and LVESV induced by lactate after MI (fig. S9, C to F), suggesting improved cardiac function. In addition, MCT1 deficiency mitigated lactate-promoted EndoMT after MI as shown in increased *Cdh5* and *Kdr* mRNA levels and decreased *Acta2*, *Fn1*, and *S100a4* mRNA levels (fig. S9, G to K). Collectively, these data indicate that extracellular lactate-promoted EndoMT is mediated by MCT-dependent mechanisms.

### Lactate promoted Snail1 nuclear translocation following hypoxia

**Fig. 8.**
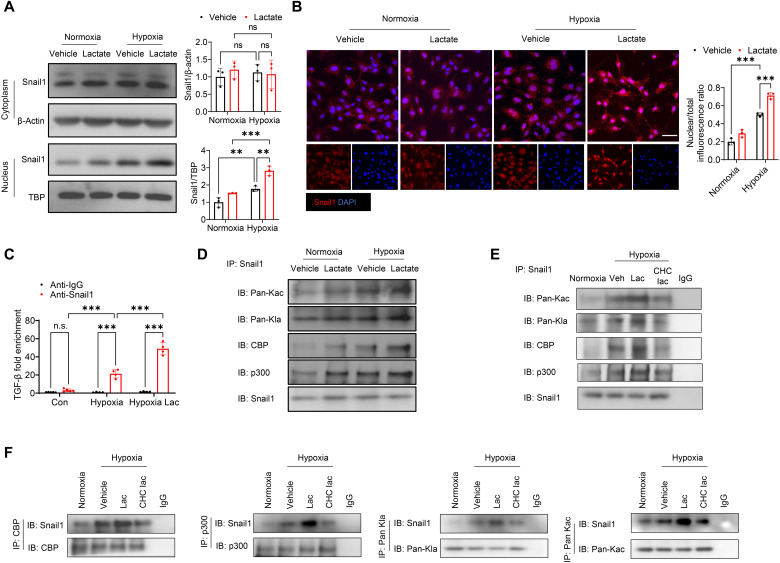
Lactate promotes Snail1 nuclear translocation and lactylation following hypoxia. HUVECs were treated with lactate (10 mM) followed by normoxic or hypoxic challenge. The expression of cytoplasmic and nuclear Snail1 expression was measured by Western blot (**A**) and immunofluorescent staining (**B**). Scale bar, 50 μm. (**C**) ChIP assay was performed with anti-Snail1 antibody followed by qRT-PCR using primers specific for TGF-β. (**D**) Immunoprecipitation (IP) was performed to examine acetylation and lactylation of Snail1, as well as the interaction between Snail1 and CBP/p300. (**E** and **F**) Endothelial cells were treated with the MCT inhibitor CHC before lactate administration. Snail1 acetylation and lactylation, and the interaction between Snail1 and CBP/p300 were measured by immunoprecipitation. *n* = 3 to 4 per group. Comparisons of data between groups were made using two-way ANOVA followed by Tukey’s procedure. **P < 0.01, ***P < 0.001 compared with indicated groups.

Snail1 is a transcriptional factor of TGF-β that plays an important role in regulating epithelial-to-mesenchymal transition (EMT) (*31*). We next investigated the role of Snail1 in lactate-induced EndoMT via TGF-β/Smad2-dependent signaling. As shown in Fig. 8A, hypoxia alone did not alter cytoplasmic expression of Snail1. However, Snail1 nuclear expression was accelerated markedly after hypoxia when compared with normoxic condition. Treatment with lactate further up-regulated nuclear translocation of Snail1. In accordance, immunofluorescent staining revealed that there was more positive staining of nuclear Snail1 following hypoxic challenge, which can be further induced by lactate administration (Fig. 8B), indicating that nuclear translocation of Snail1 may play an important role in lactate-promoted EndoMT.

We then explored whether increased Snail1 nuclear translocation could be responsible for lactate-activated TGF-β signaling. We performed chromatin immunoprecipitation (ChIP) assay using anti-Snail1 antibody and examined whether *TGFB1* gene could be detected in the immunoprecipitates with anti-Snail1 antibody. Figure 8C shows that there is a robust recruitment of Snail1 with *TGFB1* gene in hypoxia-challenged cells. However, the interaction between *TGFB1* gene and Snail1 protein was significantly greater in lactate-treated hypoxic-challenged cells than in the cells subjected to hypoxia (Fig. 8C). These data demonstrate that lactate promotes Snail1 protein and *TGFB1* gene interaction, which contributes to TGF-β/Smad2-mediated EndoMT.

### Lactate induced Snail1 lactylation after hypoxia

Epigenetics plays an important role in TGF-β–related EndoMT (*15*). A recent study clarified that lactate can induce the process of lactylation by adding a lactyl group to histone lysine (K) residues, thus regulating gene transcription (*14*). Immunoprecipitation with anti-Snail1 antibody followed by immunoblotting with anti–pan-acetyl-lysine (Kac) or anti–pan-lactyl-lysine (Kla) antibody showed that hypoxia induced acetylation and lactylation of Snail1. Administration of lactate further up-regulated Snail1 acetylation and lactylation (Fig. 8D). A previous study found that Snail is able to interact with CREB (adenosine 3′,5′-monophosphate response element–binding protein)–binding protein (CBP)/p300 to induce its acetylation (*32*). We observed that there is an interaction between Snail1 and CBP/p300 and that lactate treatment promoted their interaction after hypoxia (Fig. 8D). However, inhibition of intracellular lactate by CHC administration attenuated lactate-promoted interaction between Snail1 and CBP/p300, as well as acetylation and lactylation of Snail1 (Fig. 8E). Reciprocally, lactate treatment also significantly increased the expression of Snail1 in the immunoprecipitate with anti-CBP antibody, anti-p300 antibody, anti–pan-Kla antibody, and anti–pan-Kac antibody (Fig. 8F), when compared with the hypoxia group. In contrast, administration of CHC reduced the interaction between Snail1 and CBP/p300, and lactylation and acetylation of Snail1 (Fig. 8F).

### Silencing of Snail1 attenuated EndoMT and TGF-β/Smad2 activation after hypoxia

**Fig. 9.**
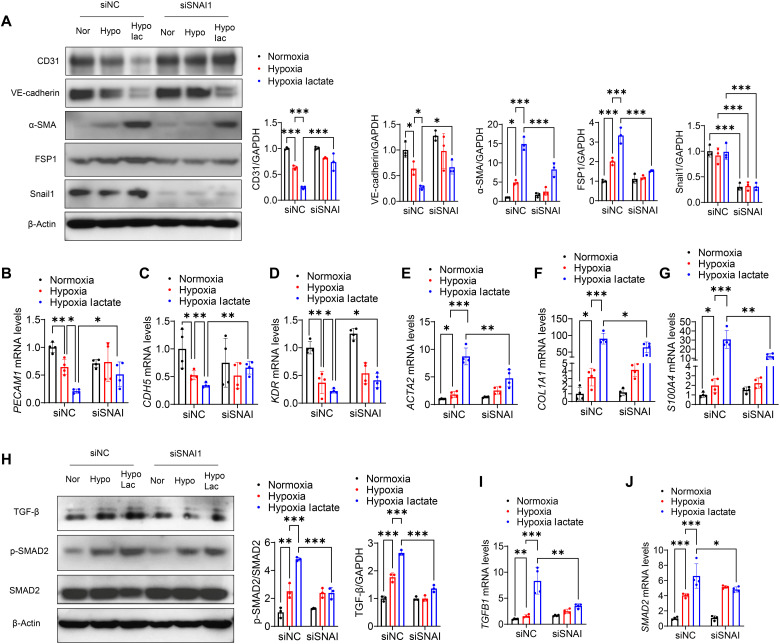
Silencing of Snail1 attenuates EndoMT and TGF-β/Smad2 activation after hypoxia. HUVECs were transfected with siRNA specific for Snail1 (siSNAI1). Scrambled siRNA served as control (siNC). Twenty-four hours after transfection, cells were treated with lactate (10 mM) followed by hypoxic challenge. The expression of endothelial markers and mesenchymal markers was measured by Western blot (**A**) and qRT-PCR (**B** to **G**). The activation of TGF-β/Smad2 was also measured by Western blot (**H**) and qRT-PCR (**I** and **J**). *n* = 3 to 4 per group. Comparisons of data between groups were made using two-way ANOVA followed by Tukey’s procedure. *P < 0.05, **P < 0.01, ***P < 0.001 compared with indicated groups.

Furthermore, we disclosed whether lactate promotes EndoMT and TGF-β/Smad2 activation through Snail1 activation. Silencing of Snail1 by transfection with its specific siRNA reversed the down-regulation of CD31 and VE-cadherin induced by lactate following hypoxia (Fig. 9A). In contrast, Snail1 inhibition prevented lactate-accelerated mesenchymal marker α-SMA and FSP1 expression (Fig. 9A), SMAD2 phosphorylation, and TGF-β activation (Fig. 9H). We also performed qRT-PCR and observed that knockdown of Snail1 enhanced the mRNA levels of *PECAM1*, *KDR*, and *CDH5* (Fig. 9, B to D) and decreased the mRNA levels of *ACTA2*, *COL1A1*, and *S100A4* (Fig. 9, E to G) in the presence of lactate treatment. Moreover, silencing of Snail1 also attenuated *SMAD2* and *TGFB1* mRNA levels induced by lactate administration (Fig. 9, I and J).

### Silencing of Snail1 improved cardiac function and decreased EndoMT after MI

**Fig. 10.**
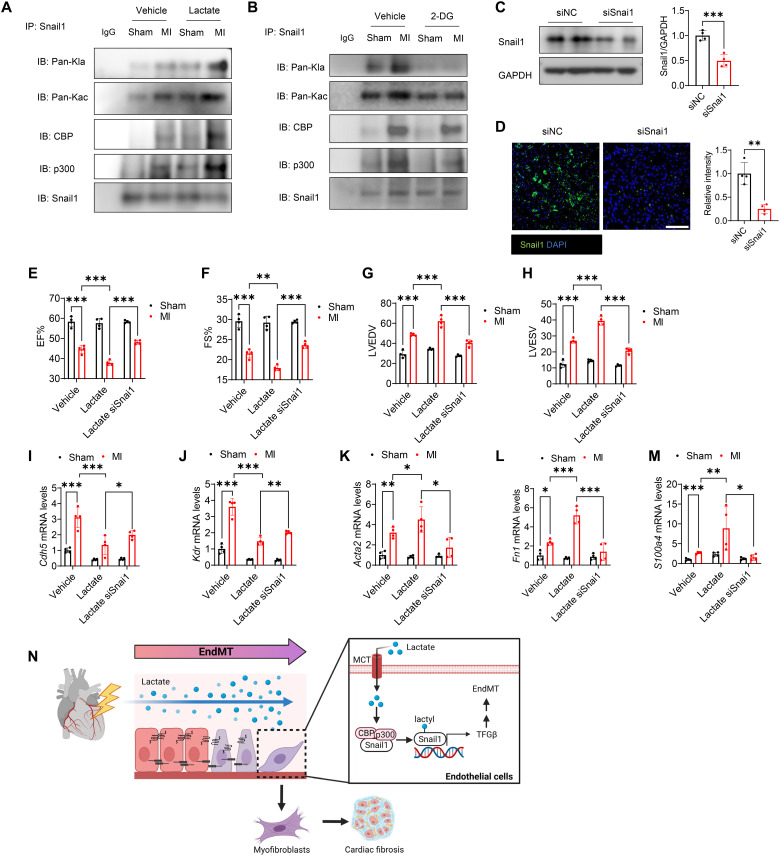
Silencing of Snail1 attenuates Snail1 lactylation and EndoMT after MI. Mice were subjected to MI or sham surgery followed by supplemental lactate or 2-DG administration. (**A** and **B**) Mouse hearts were collected and immunoprecipitation was performed to examine acetylation and lactylation of Snail1, as well as the interaction between Snail1 and CBP/p300. Mice were administered siRNA specific for Snail1 before MI or sham surgery. Western blot (**C**) and immunofluorescent staining (**D**) were performed to measure the expression of Snail1 expression in the myocardium. Scale bar, 200 μm. (**E** to **H**) Seven days after surgery, cardiac function (EF%, FS%, LVEDV, and LVESV) was measured by echo. (**I** to **M**) The mRNA levels of *Cdh5*, *Kdr*, *Acta2*, *Fn1*, and *S100a4* were examined by qRT-PCR. (**N**) Scheme of lactate-induced EndoMT after MI. Comparisons of data between groups were made using two-way ANOVA followed by Tukey’s procedure or *t* test. *P < 0.05, **P < 0.01, ***P < 0.001 compared with indicated groups.

To elucidate whether Snail1 plays a role in EndoMT in vivo, we evaluated lactylation of Snail1 in the myocardium after MI. As shown in Fig. 10 (A and B), MI significantly promoted Snail1 lactylation and acetylation, as well as the interaction between Snail1 and CBP/p300, when compared with the sham group. Lactate administration further induced their interaction (Fig. 10A). However, treatment with 2-DG attenuated MI-induced interaction between Snail1 and CBP or p300, and decreased Snail1 acetylation and lactylation (Fig. 10B). Moreover, lactate also stimulated lactylation of Snail1 in cardiac endothelial cells after MI (fig. S11A). We then transfected siRNA specific for Snail1 to knock down the expression of Snail1 in the myocardium and investigated whether it can improve cardiac function. Western blot and immunofluorescent staining confirmed that administration of siSnai1 can significantly diminish Snail1 expression in the heart (Fig. 10, C and D). Seven days after surgery, we observed that knockdown of Snail1 has no impact on cardiac function and EndoMT in sham mice (fig. S10, A to I). However, Snail1 silencing reversed lactate-decreased EF% and FS% levels (Fig. 10, E and F) and prevented LVEDV and LVESV levels induced by lactate after MI (Fig. 10, G and H), indicating that deficiency of Snail1 can improve lactate-promoted cardiac dysfunction. In addition, Snail1 silencing elevated *Cdh5* and *Kdr* mRNA expression (Fig. 10, I and J) and reduced *Acta2*, *Fn1*, and *S100a4* mRNA expression (Fig. 10, K to M) when compared with lactate treatment following MI, suggesting that Snail1 may contribute to lactate-induced EndoMT after MI. Together, our data indicate that Snail1 plays an important role in lactate-promoted EndoMT via activation of TGF-β/Smad2 signaling (Fig. 10N).

## DISCUSSION

The current study has revealed a previously unknown role of lactate in the promotion of cardiac fibrosis by regulating the process of EndoMT in the heart following MI. We demonstrated that inhibition of lactate production by 2-DG ameliorated MI-induced EndoMT, cardiac fibrosis, and cardiac dysfunction. In marked contrast, administration of supplemental lactate further promoted EndoMT and worsened cardiac dysfunction after MI. In addition, lactate treatment enhanced EndoMT after hypoxia through activation of the TGF-β/Smad2 signaling pathway. Administration of CHC, a lactate transporter MCT inhibitor, as well as silencing of MCT1 mitigated lactate-mediated EndoMT of endothelial cells. Of note, lactate accelerated the lactylation of Snail1, a transcription factor of TGF-β, promoted its nuclear translocation to bind at the promoter of TGF-β, and up-regulated TGF-β expression. Deficiency of Snail1 attenuated lactate-induced EndoMT after MI/hypoxia and improved cardiac function. Our finding suggests that lactate, as an important marker for the prognosis and mortality of heart attack and heart failure, contributes to the pathophysiology of cardiac fibrosis by promoting EndoMT following MI. Therefore, targeting lactate production and/or lactate receptor/membrane transport could be a new therapeutic approach for the patients with heart attack and heart failure. For example, approaches that enhance the clearance of excess circulating lactate, such as improvement of myocardial blood flow, could be beneficial for patients with acute MI. In addition, suppression of lactate uptake in endothelial cells by targeting MCT1 may also present a promising strategy for clinical drug development.

Lactate, considered as an end-product of anaerobic glycolysis and an important biomarker for sepsis (*33*), is produced by various tissues, such as immune cells and muscles, under pathological conditions. It is positively correlated with the prognosis and mortality of patients with heart failure (*11*, *12*). Previous clinical investigation showed that serum lactate levels in heart transplant recipient survivors are significantly lower than those in nonsurvivors, indicating that serum lactate level can be used as a biomarker to determine heart transplant recipient mortality (*34*). In accordance, Zymlinski *et al*. (*11*) observed elevated lactate levels in acute heart failure patients, which is associated with damage to the myocardium, liver, and kidney and a higher mortality rate. A recent study by Dai *et al*. (*35*) unraveled that lactate production by lactate dehydrogenase A (LDHA) promotes cardiac myocyte hypertrophy. We recently observed that extracellular lactate directly impairs endothelial cell function during sepsis/septic shock and systemic administration of lactate inhibitor attenuates endothelial cell injury during sepsis (*36*). In the present study, we demonstrate that inhibiting lactate production markedly attenuated MI-induced cardiac dysfunction and cardiac fibrosis in mice. Of note, elevated serum lactate levels by administration of supplemental lactate to MI mice exacerbated cardiac dysfunction, indicating that lactate exerts an important biological function on MI-induced cardiac fibrosis and dysfunction. However, we did not detect obvious alterations in cardiac function between vehicle and lactate-infused sham mice. One of the possibilities is that lactate is rapidly cleared by kidney or liver in sham mice.

It is well established that mesenchymal cells contribute to the development of fibroblasts and myofibroblasts in multiple organs including the heart (*37*, *38*). Cardiac fibroblasts are the main cells that are responsible for the progress of cardiac fibrosis by transforming to a myofibroblast phenotype (*39*–*41*). On the contrary, vascular endothelial cell activation protects against cardiac ischemic injury (*42*, *43*). EndoMT plays an essential role in the progress of different cardiovascular diseases such as MI, atherosclerosis, cardiac fibrosis, and valvular diseases (*37*). Recent studies have indicated that EndoMT is associated with aberrant blood vessel growth, thereby regulating cardiac remodeling after MI (*44*–*46*). Increased expression of LDHA that enhances lactate production induces EMT in tumor microenvironment and promotes tumor metastasis (*47*). Therefore, we investigated the possibility that lactate may contribute to EndoMT after MI. To investigate the possibility of lactate contributing to EndoMT after MI, we analyzed markers for endothelial cells and for mesenchymal fibroblasts after MI and found that elevated lactate levels by administration of supplemental lactate to MI mice promoted more endothelial cells transiting to mesenchymal cells in the myocardium. Consistent with a previous study (*17*), we found that although only a small percentage of endothelial cells transit to mesenchymal cells after MI, suppressed lactate production not only reduced EndoMT but also attenuated cardiac fibrosis, indicating that EndoMT may be a critical regulator of cardiac fibrosis following cardiac injury. Fibroblasts also play an important role in regulating cardiac fibrosis after MI. Whether lactate will affect cardiac fibroblast activation directly remains unclear and needs to be clarified in the future. Similar results were observed in in vitro studies, showing that treatment of endothelial cells with lactate decreased endothelial cell marker VE-cadherin expression and increased fibroblast marker α-SMA expression in endothelial cells following hypoxia. Our finding suggests that lactate increases cardiac fibrosis by promoting EndoMT in the myocardium after MI.

Besides its function in regulating fibrosis in multiple fibrotic diseases, TGF-β is also a primary factor in driving the process of EndoMT (*48*, *49*). The TGF-β signaling component Smad2/3 plays an essential role in activating fibroblasts in the heart (*50*). Deficiency of either Smad2 or Smad3 ameliorates myofibroblast activation (*50*). In the present study, we found that treatment of lactate enhanced TGF-β and Smad2 activation, but not Smad3 expression and phosphorylation, both in vivo and in vitro. Our data indicate the involvement of TGF-β/Smad2 signaling in lactate-stimulated EndoMT after MI/hypoxia. Extracellular lactate can be released though MCTs, contributing to the increased lactate levels in the intracellular environment (*29*, *51*). In our study, we observed that both pharmacological and genetic suppression of MCT1 can attenuate lactate-induced EndoMT and TGF-β/Smad2 activation in endothelial cells. According to the Human Protein Atlas, MCT1 is expressed in endothelial cells, cardiac myocytes, and fibroblasts. Although cardiomyocytes have the highest MCT1 level, the MCT1 expression level in endothelial cells is two times higher than that in fibroblasts (*52*). MCT1 deficiency in the myocardium ameliorates cardiac dysfunction and EndoMT in response to lactate administration after MI. Our finding suggests that MCTs play an important role in mediating the transportation of the extracellular lactate into the intracellular environment for activation of TGF-β/Smad2 signaling, resulting in promoting EndoMT. However, as a limitation, we cannot exclude the effects of endogenous lactate produced by endothelial cells on EndoMT in this study.

Naber *et al*. (*53*) found that Snail1, a transcriptional factor of TGF-β, contributes to TGF-β–promoted EMT. According to the Human Protein Atlas, the levels of Snail1 expression in cardiac endothelial cells are significantly greater than in other cardiac cell types, including cardiac fibroblasts and cardiomyocytes (*52*). In addition, EMT induces nuclear translocation of Snail1 and silencing of Snail1 attenuates hypoxia-stimulated EMT (*54*). A recent study showed that Snail-mediated EndoMT contributes to aberrant vascularization and impaired cardiac repair after MI (*46*). In the current study, we observed that lactate administration promoted Snail1 nuclear translocation following hypoxia. ChIP assay demonstrated that nuclear Snail1 binds to TGFB1 gene to promote TGF-β expression. Moreover, inhibition of Snail1 suppressed EndoMT and TGF-β/Smad2 activation in the presence of lactate after hypoxia, showing the effect of Snail1 in lactate-induced TGF-β/Smad2-mediated EndoMT. In vivo, silencing of Snail1 improves cardiac dysfunction and EndoMT stimulated by lactate following MI. Recent studies revealed that lactate works as a potent signaling molecule and an epigenetic regulator that directly regulate gene transcription (*6*, *14*, *55*, *56*). This investigation indicates that intracellular lactate plays a critical role in the regulation of epigenetics, which could be an important mechanism of EndoMT. As histone acetylases, CBP/p300 has been reported to interact with Snail1 to induce Snail1 acetylation (*32*, *57*). We found that lactate treatment stimulated not only acetylation of Snail1 but also lactylation of Snail1 after hypoxia/MI. Administration of the MCT inhibitor CHC mitigated Snail1 acetylation as well as lactylation induced by lactate. Together, our finding suggests that lactate-induced Snail1 lactylation and nuclear translocation is an important mechanism of lactate-promoted EndoMT via activation of TGF-β/Smad2 signaling following MI. In addition to our finding, Zhang *et al*. (*14*) demonstrated that histone lactylation is induced by hypoxia. Therefore, it is possible that histone lactylation may play a similar role in lactate-induced EndoMT after MI/hypoxia.

In summary, our study uncovered a previously unrecognized role of lactate in promoting EndoMT after MI/hypoxia. Lactate induces the lactylation and nuclear translocation of Snail1, thereby regulating EndoMT by activating the TGF-β/Smad2 signaling pathway. This finding provides previously unknown insight that lactate exerts an important biological function, contributing to MI-increased cardiac fibrosis and worsened cardiac dysfunction.

## MATERIALS AND METHODS

### Experimental animals

Both wild-type (WT) C57BL/6 mice and endothelial cell–specific GFP-labeled (TIE2GFP) mice were purchased from The Jackson Laboratory (Indianapolis, IN). Mice were maintained and bred in the Division of Laboratory Animal Resources at East Tennessee State University (ETSU). All experimental procedures were performed in accordance with the *Guide for the Care and Use of Laboratory Animals* published by the National Institutes of Health (NIH Publication, 8th edition, 2011) and approved by the ETSU Committee on Animal Care.

### Induction of MI injury and echocardiography

Sex- and age-matched 8- to 10-week-old mice were ventilated by a rodent ventilator (Hugo Sachs Elektronik, March, Germany), kept anesthetizing by inhalation of 1.5 to 2% isoflurane (Henry Schein Animal Health, OH, USA) driven by 100% oxygen flow, and placed on a 37°C heating pad. Mice were subjected to MI surgery as described previously by our group (*58*). In brief, after a left thoracotomy between the third and fourth intercostal space, the heart was exposed and the left anterior descending coronary artery was permanently ligated with an 8-0 silk ligature. Sham surgically operated mice served as sham control. Mice assigned to the treated groups were given 2-DG at 0.5 g/kg body weight daily for 7 days beginning 1 day before surgery by intraperitoneal injection. In separate experiments, supplemental lactic acid (adjust to pH 6.8, 0.5 g/kg body weight; Sigma-Aldrich) was administrated to mice either by intraperitoneal injection every 7 days for 1 or 4 weeks or using osmotic mini-pumps (0.25 μl per hour; Alzet, CA, USA).

Cardiac function was measured 7 or 28 days after sham or MI surgery by echocardiography with the Toshiba Medical Aplio 80 Imaging System (Tochigi, Japan). Transthoracic two-dimensional M-mode Doppler spectral tracings were used to measure LV wall thickness, LV end-diastolic diameter, and LV end-systolic diameter. EF% and FS% were calculated as previously described (*59*, *60*).

### In vivo transfection of siRNA into mouse heart

To knock down the expression of Snail1 or MCT1 in the myocardium, Invivofectamine 3.0 reagent (IVF-3005, Invitrogen by Life Technologies, CA, USA) and siSnai1 (to knock down the expression of Snail1) or siSlc16a1 (to knock down the expression of MCT1) were mixed and incubated at 55°C for 30 min. After incubation, a total volume of 200 μl of siRNA was injected through the right common carotid artery with a microcatheter as described in our previous study (*59*). The expression of Snail1 or MCT1 in the heart was measured by Western blot and immunofluorescent staining.

### Masson’s trichrome staining

Mouse hearts were harvested, cut horizontally, fixed in 4% buffered paraformaldehyde, embedded in paraffin, and cut at a thickness of 5 mm. The cardiac sections were stained with trichrome stain (Masson) kit (Sigma-Aldrich, MO, USA), mounted with synthetic resin (Vector Laboratories, CA, USA) in accordance with the manufacturer’s protocol (*61*), and examined with the EVOS Microscope (Thermo Fisher Scientific, MA, USA).

### 2,3,5-Triphenyltetrazolium chloride staining

Infarct size was evaluated by 2,3,5-triphenyltetrazolium chloride (TTC) staining as previously described (*59*, *62*). Briefly, 7 days after sham or MI surgery, hearts were collected and cut into five slices followed by staining with 0.8% TTC solution (LABLEAD, Beijing, China, 0765) at 37°C for 15 min.

### Apoptosis assay

Myocardial apoptosis was measured by TUNEL staining. The cardiac sections were stained with the One Step TUNEL Apoptosis Assay Kit (Beyotime, Shanghai, China, C1088) according to the manufacturer’s protocol. The apoptotic rate was calculated by normalizing the number of TUNEL-positive cells to the 4′,6-diamidino-2-phenylindole (DAPI)–stained cells.

### Lactate measurement

Mouse serum was isolated from the supernatant of clotted whole blood by centrifuging at 2500*g* at 4°C for 10 min. Serum, heart tissue, and cell lactate levels were measured by Lactate Assay Kit II (Sigma-Aldrich, MO, USA) according to the manufacturer’s protocol. For serum samples, a 0.5-μl serum sample was diluted in 49.5 μl of lactate assay buffer, mixed with 50 μl of reaction mixes using a horizontal shaker, and incubated at room temperature for 30 min, and the absorbance (450 nm) was examined. For heart tissue samples, samples were homogenized in four volumes of the lactate assay buffer and centrifuged. The soluble fraction was ready to be assayed. To prepare cell samples, cells were homogenized in four volumes of lactate assay buffer, centrifuged, and deproteinized with a 10-kDa molecular weight cutoff spin filter.

### Flow cytometry

To detect the process of EndoMT, heart tissues from treated TIE2GFP mice were digested with digestion buffer [collagenase I (1 mg/ml) and collagenase IV (1 mg/ml)] at 37°C. Cell suspensions were stained with single-color antibodies [anti-CD31 (BD Biosciences, 561410), anti-CD144 (BD Biosciences, 562242), and anti-gp38 (BioLegend, 127407)] for 30 min on ice, washed, and examined using a BD FACS Fortessa flow cytometer (Becton Dickinson, NJ, USA). Results were analyzed with the FlowJo software.

### Isolation of mouse cardiac endothelial cells

Endothelial cells were isolated from mouse hearts as previously described (*63*). Briefly, the day before cell harvest, 200 μl of anti-rat immunoglobulin G (IgG) Dynabeads (Invitrogen, #11035) was incubated with 20 μl of CD31 antibody (BioLegend, #102504) at 4°C with constant stirring. Heart tissues were minced and digested in collagenase I (1 mg/ml; Thermo Fisher Scientific, #17100-017) and collagenase II (1 mg/ml; Thermo Fisher Scientific, #17101-015) in Dulbecco’s modified Eagle’s medium for 20 min at 37°C water bath. Cell suspension was filtered through a 40-μm strainer and incubated with CD31-coated beads (25 μl per heart) for 15 min at room temperature. Beads were washed on a magnetic column for five times. Isolated primary cardiac endothelial cells were used for the following analysis.

### Cell culture

HUVEC cell line was purchased from the American Type Culture Collection (ATCC) (VA, USA) and cultured in Vascular Cell Basal Medium (ATCC) supplemented with growth factors (Endothelial Cell Growth Factor Kit-VEGF, ATCC) and 5% fetal bovine serum (FBS). HCMECs were purchased from ScienCell (CA, USA) and cultured in endothelial cell medium with 5% FBS, endothelial cell growth supplement, and antibiotic solution (#1001, ScienCell). Endothelial cells were treated with 5 or 10 mM l-lactic acid (Sigma-Aldrich) followed by normoxic or hypoxic challenge in a hypoxia chamber (ProOx Model C21, BioSpherix Ltd., Redfield, NY, USA) at 37°C with 5% CO_2_ and 0.1% O_2_ for 72 hours. In separate experiments, when HUVECs reached 70 to 80% confluence, they were transfected with siRNA (80 nM; Invitrogen by Life Technologies, CA, USA) specific for Snail1 (siSNAI1) or MCT1 (siSLC16A1). Scrambled siRNAs served as controls. Twenty-four hours after transfection, cells were treated with or without lactate and subjected to hypoxia.

### Cell migration assay

Endothelial cell migration was determined by wound-healing (or scratch) assay as mentioned in our previous study (*58*). HUVECs were scratched with 200-μl tips when cells reached 80% confluence, incubated with empty medium without growth factor after scratching, and photographed 16 hours after injury. The percent closure of the wound was analyzed by ImageJ (NIH).

### Proliferation assay

Endothelial cell proliferation was evaluated 16 hours after normoxic or hypoxic stimulation by EdU incorporation assay (Click-iT EdU Imaging Kit, Invitrogen by Life Technologies) as described in our previous study (*58*). The proliferation rate of EdU staining was calculated by normalizing the number of EdU-positive cells to the Hoechst-stained cells.

### Matrigel-based angiogenesis assay

Endothelial cell angiogenesis was measured with the Matrigel-based angiogenesis assay (*58*, *64*). After treatment of HUVECs as described above, the cells were seeded on Matrigel-coated 96-well plates (Corning, NY, USA) with 10^4^ cells per well and photographed 6 hours later. The total number of master junction was quantified by ImageJ (NIH).

### Collagen gel contraction assay

Collagen gel contraction assay was performed to assess cell fibroblast-like function (*65*). Briefly, processed HUVECs were mixed with collagen I solution on ice, and the mixture was added to 24-well plates (Corning, NY, USA) with 10^5^ cells per well. After incubating at 37°C with 5% CO_2_ for 20 min, the mixture was solidified and serum-free medium was added. The gel area was measured 6 hours after gel detachment using ImageJ software.

### Immunofluorescent staining

For in vivo studies, paraffin-embedded heart tissue sections were deparaffinized and rehydrated, blocked in 10% bovine serum albumin (BSA) (Thermo Fisher Scientific, MA, USA), and incubated with primary antibodies, specific anti-GFP antibody (1:100 dilution; ABclonal, AE011), anti-FSP1 antibody (1:50 dilution; Abcam, ab93283), anti-Snail1 antibody (1:100 dilution; eBioscience, 14-9859-82), and anti-MCT1 antibody (1:100 dilution; ABclonal, A9061) at 4°C overnight. For in vitro studies, endothelial cells were fixed with 3.7% formaldehyde (Sigma-Aldrich, MO, USA), permeabilized with 0.1% Triton X-100 (Sigma-Aldrich, MO, USA) in phosphate-buffered saline (PBS), and blocked with 3% BSA in PBS followed by incubating with primary antibodies at 4°C overnight. The following primary antibodies were used for immunofluorescent staining: anti–VE-cadherin antibody (1:50 dilution; Abcam, ab33168), anti-CD31 antibody (1:50 dilution; Abcam, ab28364), anti-FSP1 antibody (1:50 dilution; Abcam, ab93283), and anti-Snail1 antibody (1:100 dilution; eBioscience, 14-9859-82). The next day, slides or cells were stained with secondary antibodies at room temperature for 90 min and mounted in mounting medium with DAPI (Vector Laboratories, CA, USA).

### Western blot

Protein levels of heart tissues or endothelial cells were measured by Western blot as described (*66*). The following primary antibodies were used: anti–VE-cadherin antibody (1:1000 dilution; Abcam, ab33168), anti-CD31 antibody (1:1000 dilution; Abcam, ab28364), anti-FSP1 antibody (1:500 dilution; Abcam, ab93283), anti–α-SMA antibody (1:1000 dilution; Cell Signaling Technology, 48938S), anti-Collagen1a1 antibody (1:1000 dilution; Cell Signaling Technology, 91144S), anti–β-actin antibody (1:5000 dilution; Invitrogen, MA1-140), anti–TGF-β antibody (1:1000 dilution; Cell Signaling Technology, 3711S), anti–glyceraldehyde-3-phosphate dehydrogenase (GAPDH) antibody (1:2000 dilution; Invitrogen, MA5-15738), anti–p-SMAD2 antibody (1:1000 dilution; Cell Signaling Technology, 18338T), anti–p-SMAD3 antibody (1:1000 dilution; Cell Signaling Technology, 9520T), anti-SMAD2/3 antibody (1:1000 dilution; Cell Signaling Technology, 8685T), anti-Snail1 antibody (1:500 dilution; eBioscience, 14-9859-82), anti-Histone3 antibody (1:2000 dilution; Novus Biologicals, nb500-171), anti-TBP antibody (1:1000 dilution; Abcam, ab51841), anti-Kla antibody (1:1000 dilution; PTM BIO, PTM-1401), anti-SMAD2 antibody (1:1000 dilution; Cell Signaling Technology, 5339T), and anti-MCT1 antibody (1:1000 dilution; ABclonal, A9061). The signals were analyzed and quantified using the G:Box gel imaging system (Syngene, MD, USA).

### Quantitative real-time PCR

Total RNA was isolated from heart tissues, HCMEC, and HUVECs using RNAzol RT (Molecular Research Center, OH, USA) according to the manufacturer’s protocol as described previously (*58*). mRNA was converted to cDNA by the High-Capacity cDNA Transcription Kit (Applied Biosystems, CA, USA), and qRT-PCR was performed using SYBR Green SuperMix (Sigma-Aldrich, MO, USA) and specific primers (Sigma-Aldrich, MO, USA) on a 4800 RT-PCR machine (Bio-Rad, CA, USA), with the thermal cycler conditions suggested by the manufacturer. The mRNA levels were quantified with the 2^−ΔΔCT^ relative quantification method that were normalized to *ACTB* or *Actb*. The information of primers can be found in table S1.

### Immunoprecipitation

Immunoprecipitation was performed as described in our previous studies (*6*, *58*). Briefly, about 200 μg of protein of HUVECs, heart tissue, or cardiac endothelial cells was incubated with 2 μg of anti-Snail1, anti–pan-Kla, anti–pan-Kac, anti-CBP, or anti-p300 antibody overnight at 4°C followed by adding 20 μl of protein A/G agarose beads (Santa Cruz Biotechnology) and incubating for another 4 hours. We then washed the precipitates and boiled them in SDS sample buffer. The supernatant was subjected to immunoblotting with anti–pan-Kla antibody, anti–pan-Kac antibody (1:1000 dilution; ABclonal, A2391), anti-CBP antibody (1:1000 dilution; Cell Signaling Technology, 7389S), anti-p300 antibody (1:1000 dilution; Cell Signaling Technology, 86377S), or anti-Snail1 antibody (1:500 dilution; eBioscience, 14-9859-82), respectively.

### ChIP-qPCR

ChIP assay was performed according to the manufacturer’s protocol as we described previously (*58*). qPCR was performed using SYBR Green ReadyMix (MilliporeSigma, MA, USA) and conducted using a 4800 RT-PCR machine (Bio-Rad, CA, USA). The forward primer of TGF-β for Snail1 is 5′-CCACGTAGTACACGATGGGC-3′; the reverse primer is 5′-CGGGGAGAGACGAAGTGAGA-3′.

### Statistics

All the data are presented as means ± SD. Two-tailed Student’s *t* test or two-way analysis of variance (ANOVA) followed by Tukey’s procedure was performed to compare data among groups. *P* < 0.05 was considered to be significant.

## Supplementary Material

20230203-1

20230203-2

## Notes

View/request a protocol for this paper from *Bio-protocol*.
