# Supplementary material for: Lactate promotes endothelial-to-mesenchymal transition via Snail1 lactylation after myocardial infarction
Source: Sci Adv. 2023 Feb 3;9(5):eadc9465. doi: 10.1126/sciadv.adc9465 (PMC9897666; doi:10.1126/sciadv.adc9465)
Supplement: Supplementary file 3 — Figs. S1 to S11 Table S1 [file sciadv.adc9465_sm.pdf]

Science Advances
AAAS

Supplementary Materials for

Lactate promotes endothelial-to-mesenchymal transition via Snail1 lactylation after myocardial infarction

Min Fan *et al.*

Corresponding author: Chuanfu Li, li@etsu.edu

Sci. Adv. **9**, eadc9465 (2023)
DOI: 10.1126/sciadv.adc9465

This PDF file includes:

Figs. S1 to S11
Table S1

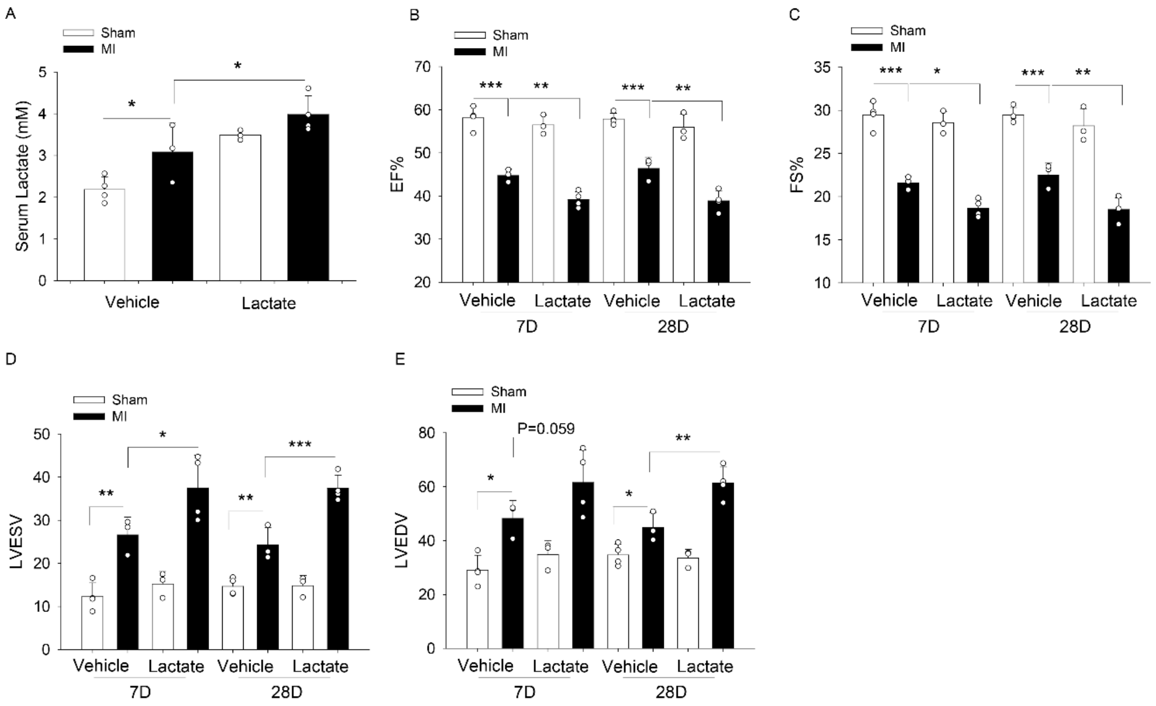

Supplemental Figure 1. Increased lactate levels result in worsened cardiac dysfunction after myocardial infarction (MI). Mice were subjected to MI or sham surgery followed by supplemental lactate or vehicle administration using osmotic mini-pumps (0.25 μL/hour). (**A**) Three days after surgery, serum lactate levels were measured by commercially available kit. (**B-E**) Left ventricular ejection fraction (EF%), fractional shortening (FS%), left ventricular end diastolic volume (LVEDV) and LV end systolic volume (LVESV) were tested 7 and 28 days after MI or sham surgical operation. n=3-4/group. Comparisons of data between groups were made using 2-way ANOVA followed by Tukey's procedure. *P < 0.05, **P < 0.01, ***P < 0.001 compared with indicated groups.

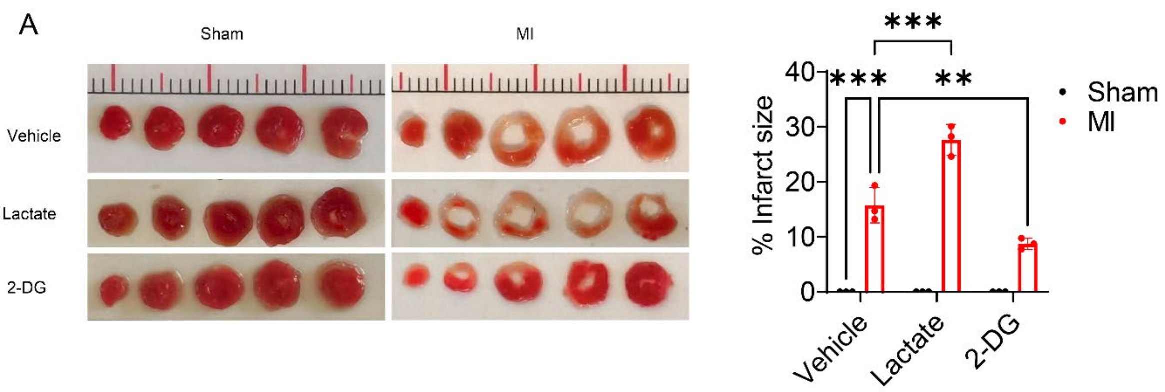

Supplemental Figure 2. Increased lactate levels result in induced infarct size after MI. Mice were subjected to MI or sham surgery followed by supplemental lactate, 2-DG or vehicle administration. (**A**) TTC staining was performed to measure cardiac infarct size. n=3/group. Comparisons of data between groups were made using 2-way ANOVA followed by Tukey's procedure. **P < 0.01, ***P < 0.001 compared with indicated groups.

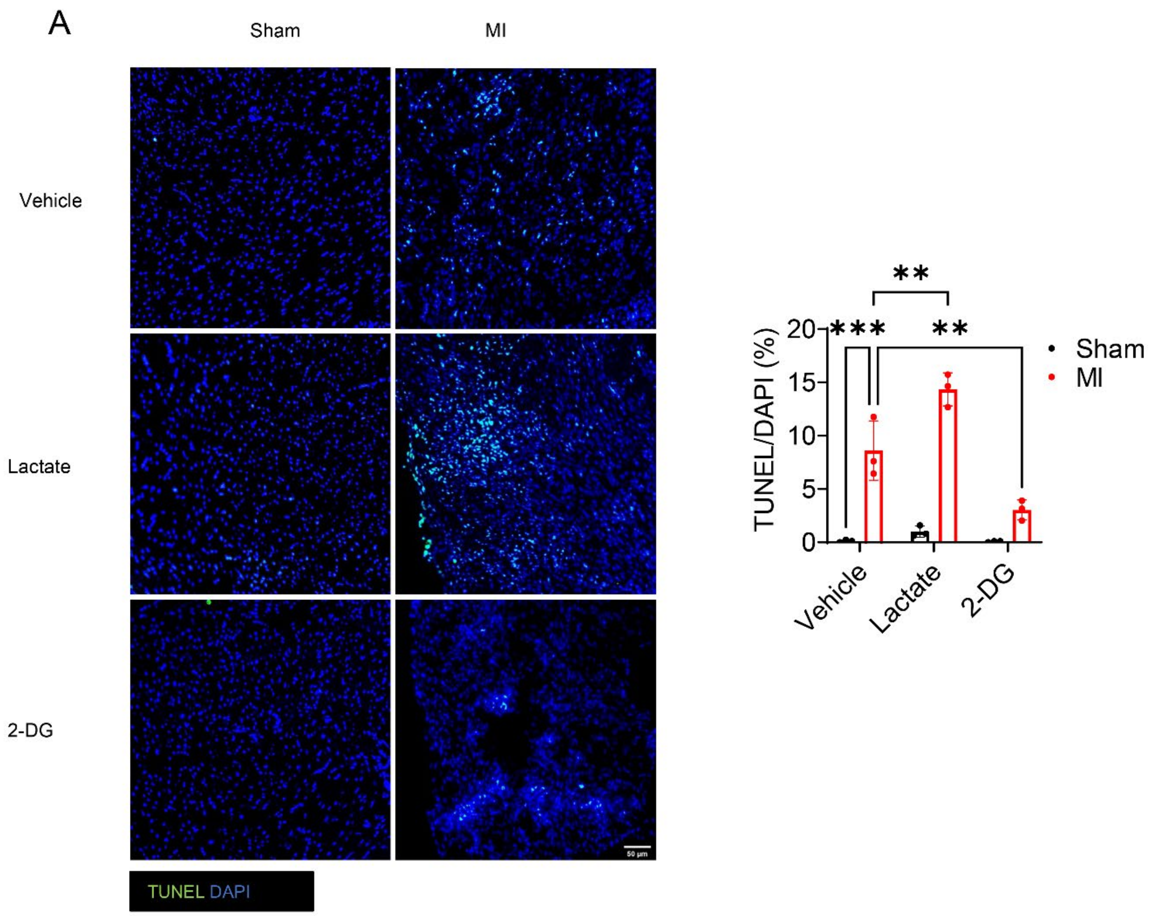

Supplemental Figure 3. Increased lactate levels accelerate cardiac apoptosis after MI. Mice were subjected to MI or sham surgery followed by supplemental lactate, 2-DG or vehicle administration. (**A**) TUNEL staining was performed to measure cardiac apoptosis. n=3/group. Comparisons of data between groups were made using 2-way ANOVA followed by Tukey's procedure. **P < 0.01, ***P < 0.001 compared with indicated groups.

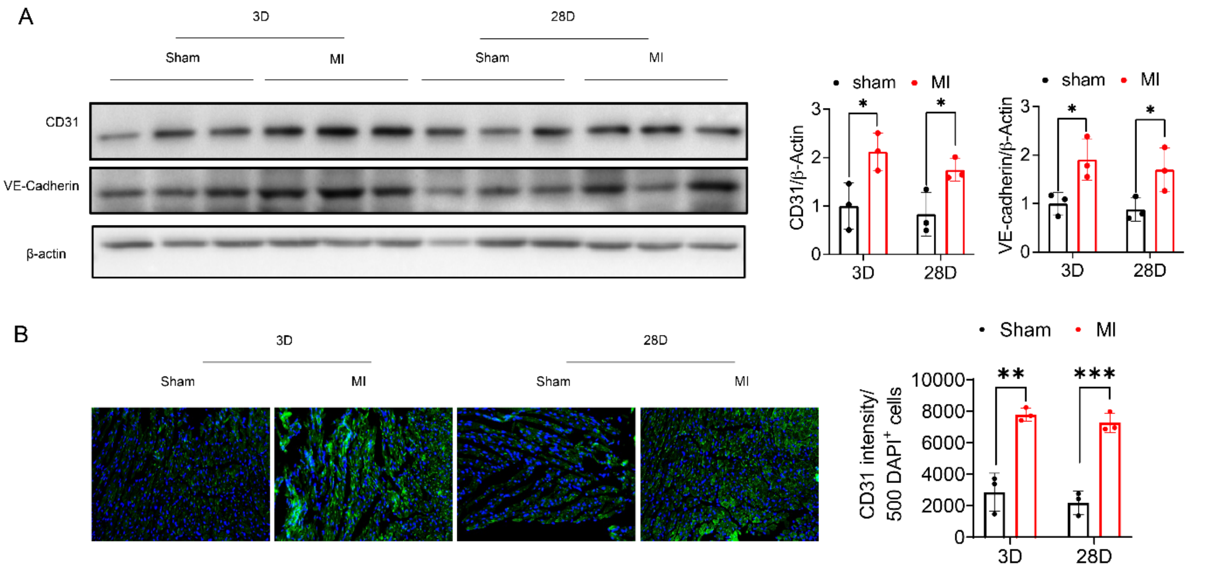

Supplemental Figure 4. Myocardial infarction induces CD31 and VE-Cadherin expression in the myocardium. Mice were subjected to MI or sham surgery. The expression of cardiac CD31 was measured by Western Blot (**A**) and immunofluorescent staining (**B**). The expression of cardiac VE-Cadherin was measured by Western Blot (**A**). n=3/group. Comparisons of data between groups were made using unpaired t-test**.** *P < 0.05, **P < 0.01, ***P < 0.001 compared with indicated groups.

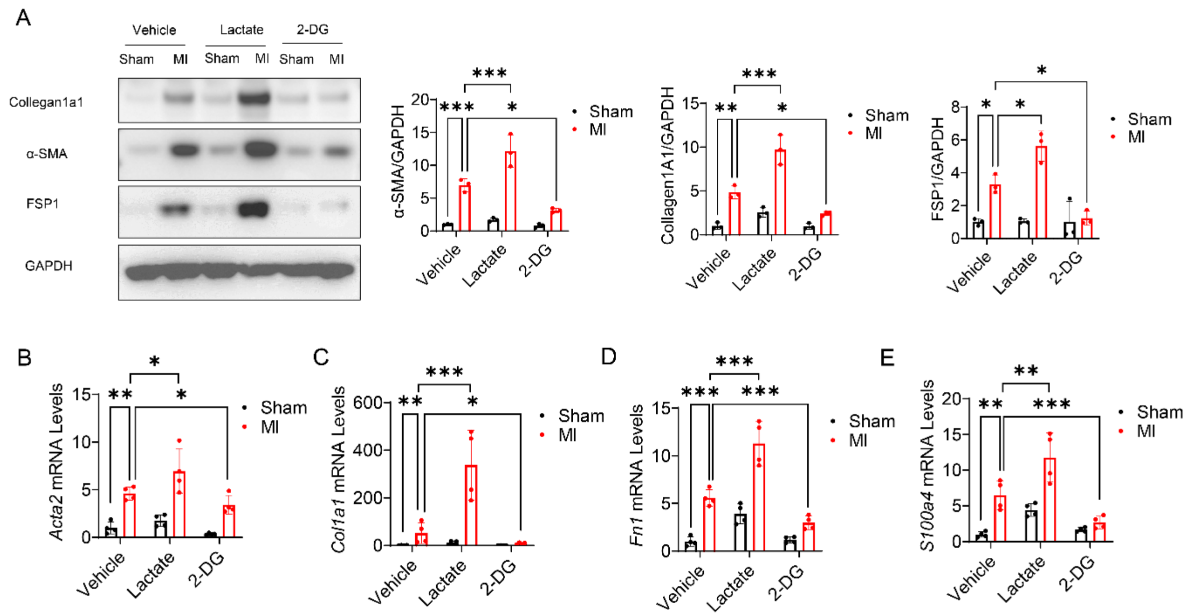

Supplemental Figure 5. Increased lactate levels promote mesenchymal marker expression in cardiac endothelial cells after MI. Mice were subjected to MI or sham surgery followed by supplemental lactate, 2-DG or vehicle administration. Cardiac endothelial cells were isolated from the hearts. The expression of mesenchymal markers was measured by Western Blot (**A**) and qRT-PCR (**B-E**). n=3-4/group. Comparisons of data between groups were made using 2-way ANOVA followed by Tukey's procedure. *P < 0.05, **P < 0.01, ***P < 0.001 compared with indicated groups.

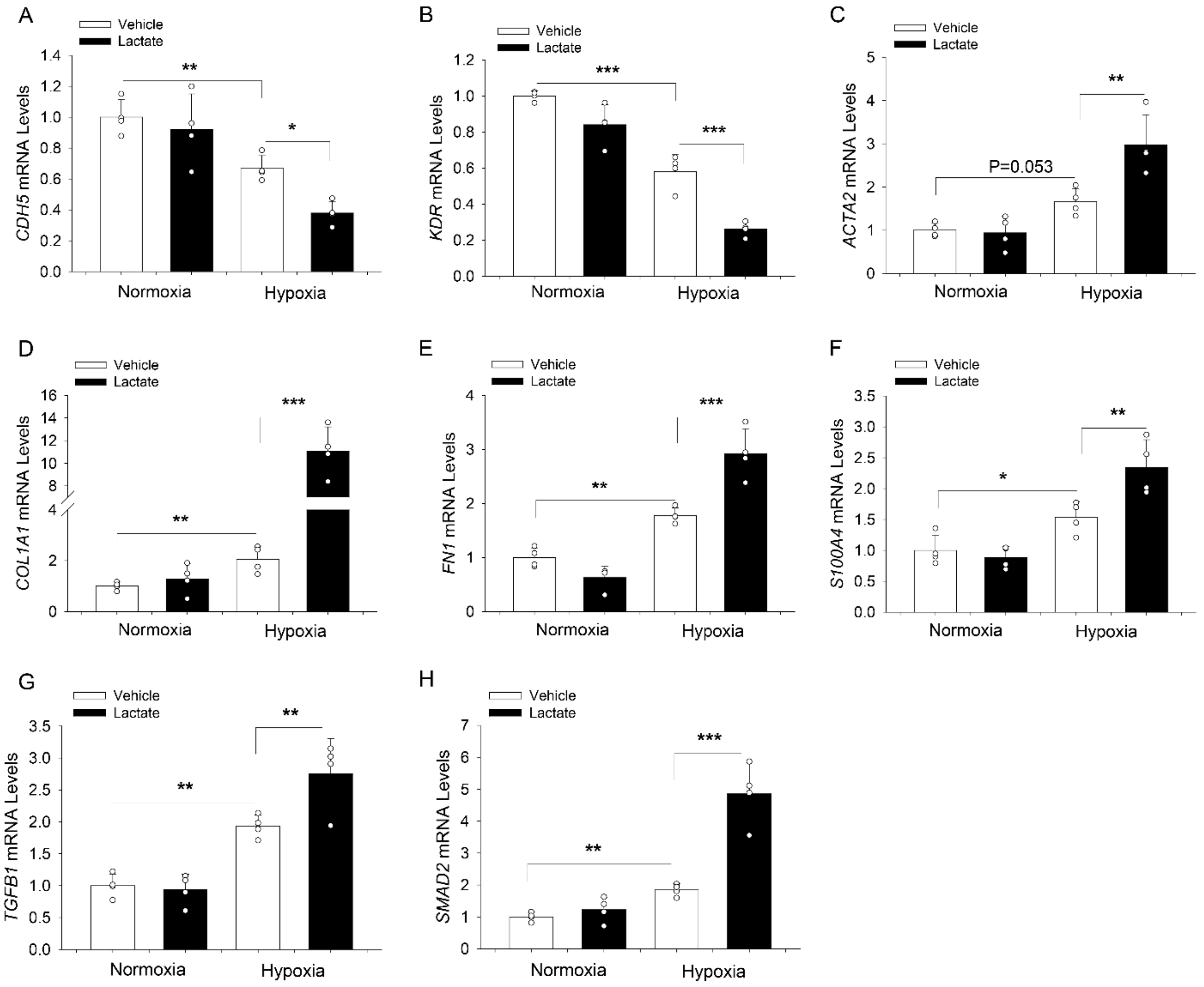

Supplemental Figure 6. Lactate promotes EndoMT in endothelial cells after hypoxia. HCMECs were treated with lactate (10 mM) followed by normoxic or hypoxic challenge. The mRNA expression of EndoMT markers (**A-F**) and *TGFB1*/*SMAD2* (**G-H**) were examined by qRT-PCR. n=3-4/group. Comparisons of data between groups were made using 2-way ANOVA followed by Tukey's procedure. *P < 0.05, **P < 0.01, ***P < 0.001 compared with indicated groups.

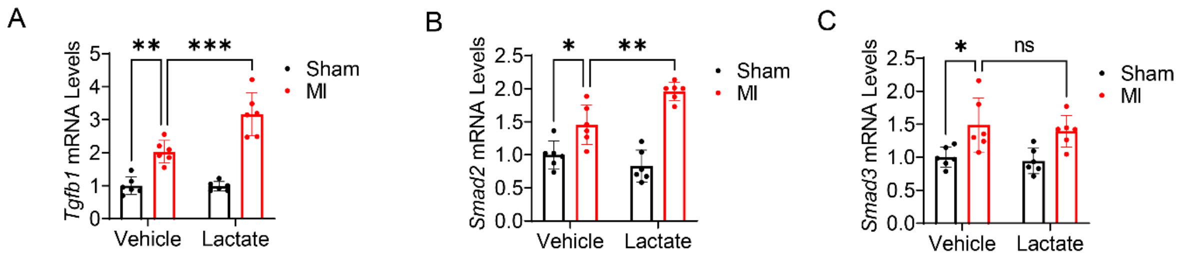

Supplemental Figure 7. Lactate stimulates TGF-β/Smad2 signaling after MI in cardiac endothelial cells. Mice were subjected to MI or sham surgery followed by supplemental lactate or vehicle administration. Cardiac endothelial cells were isolated from the hearts. (**A-C**), The expression of *Tgfb1*, *Smad2* and *Smad3* mRNA levels were measured by qRT-PCR. n=6/group. Comparisons of data between groups were made using 2-way ANOVA followed by Tukey's procedure. *P < 0.05, **P < 0.01, ***P < 0.001 compared with indicated groups.

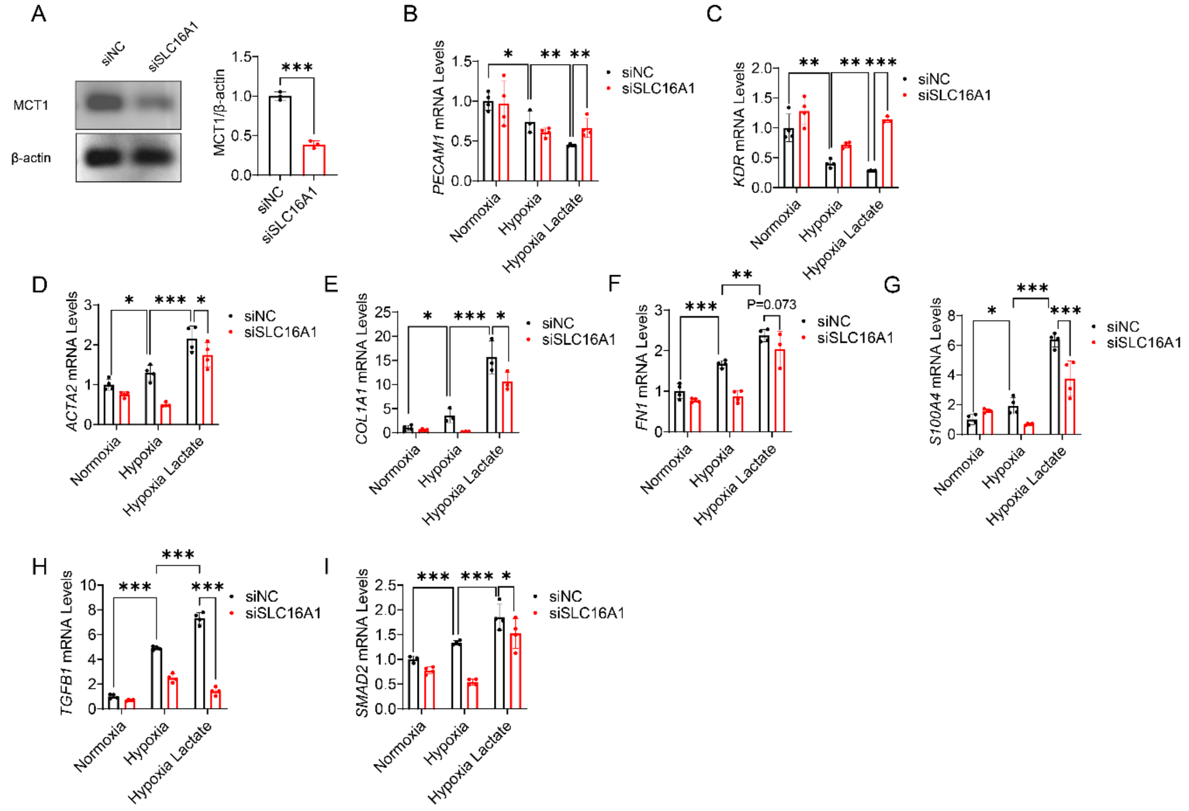

Supplemental Figure 8. Silencing of MCT1 attenuates lactate induced EndoMT and TGF-β/Smad2 activation after hypoxia. Endothelial cells were treated with siRNA for MCT1 prior to lactate administration. (**A**) The expression of MCT1 was measured by Western Blot. (**B-I**) The mRNA levels of *PECAM1*, *KDR*, *ACTA2*, *FN1*, *COL1A1*, *S100A4*, *TGFB1* and *SMAD2* were examined by qRT-PCR. n=3-4/group. Comparisons of data between groups were made using 2-way ANOVA followed by Tukey's procedure or t-test. *P < 0.05, **P < 0.01, ***P < 0.001 compared with indicated groups.

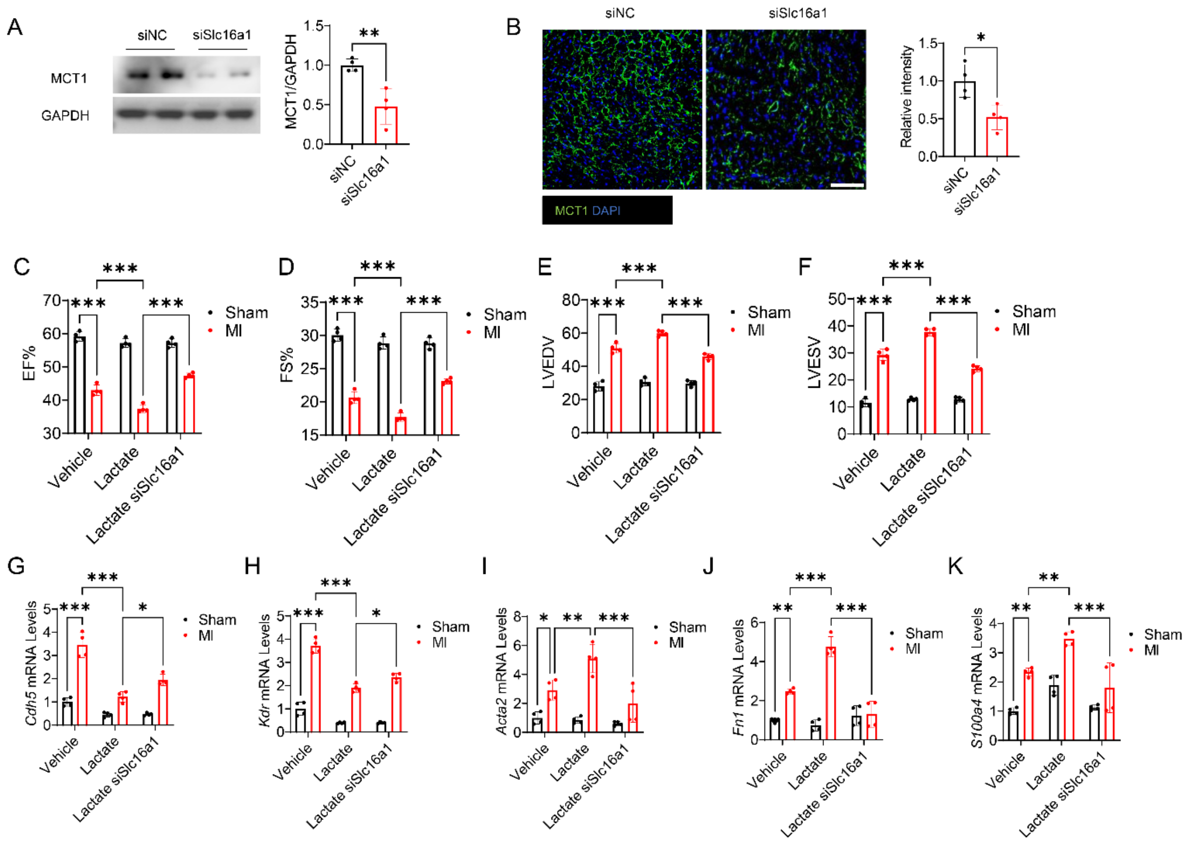

Supplemental Figure 9. Silencing of MCT1 attenuates lactate induced EndoMT after MI. Mice were administrated with siRNA specific for MCT1 before MI or Sham surgery. Western Blot (**A**) and immunofluorescent staining (**B**) were performed to measure the expression of Snail1 expression in the myocardium. Scale bar: 100μm. (**C-F**) Seven days after surgery, cardiac function (EF%, FS%, LVEDV and LVESV) was measured by echo. (**G-K**) The mRNA levels of *Cdh5*, *Kdr*, *Acta2*, *Fn1* and *S100a4* were examined by qRT-PCR. n=3-4/group. Comparisons of data between groups were made using 2-way ANOVA followed by Tukey's procedure or t-test. *P < 0.05, **P < 0.01, ***P < 0.001 compared with indicated groups.

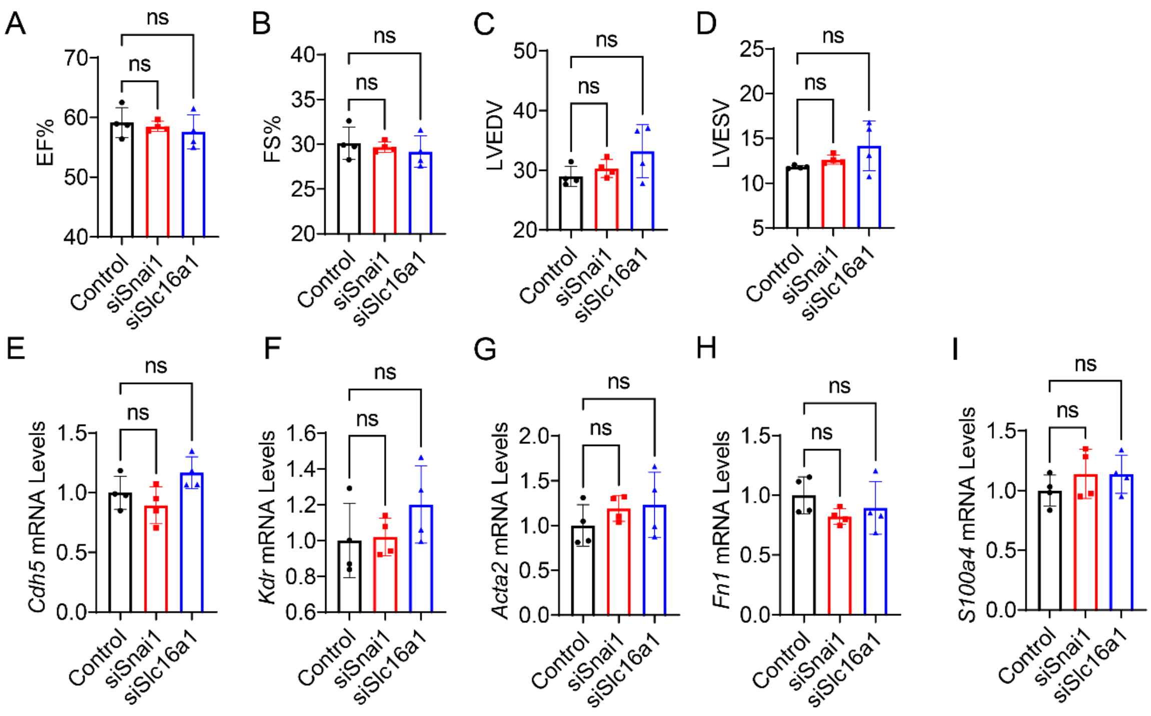

Supplemental Figure 10. Silencing of either Snail1 or MCT1 alone does not alter cardiac function and EndoMT. Mice were administrated with siRNA specific for Snail1 or MCT1. (**A-D**) Seven days after surgery, cardiac function (EF%, FS%, LVEDV and LVESV) was measured by echo. (**E-I**) The mRNA levels of *Cdh5*, *Kdr*, *Acta2*, *Fn1* and *S100a4* were examined by qRT-PCR. n=4/group. Comparisons of data between groups were made using 1-way ANOVA followed by Tukey's procedure.

A

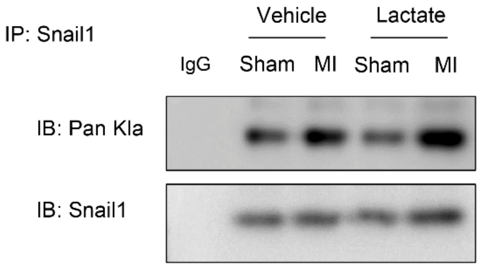

Supplemental Figure 11. Lactate promotes Snail1 lactylation in cardiac endothelial cells after MI. Mice were subjected to MI or sham surgery followed by supplemental lactate or vehicle administration. Cardiac endothelial cells were isolated from the hearts. (**A**) Immunoprecipitation was performed to examine lactylation of Snail1.

Supplemental Table 1. Primers used for qRT-PCR analysis.

Names	Sequences (5'->3')
Acta2 Forward Primer	CATCTTTCATTGGGATGGAG
Acta2 Reverse Primer	TTAGCATAGAGATTCTTCCTG
Col1a1 Forward Primer	CGTATCACCAAACTCAGAAG
Col1a1 Reverse Primer	GAAGCAAAGTTTCCTCCAAG
Fn1 Forward Primer	CCTATAGGATTGGAGACACG
Fn1 Reverse Primer	GTTGGTAAATAGCTGTTCGG
S100a4 Forward Primer	TATTCAGCACTTCCTCTCTC
S100a4 Reverse Primer	CCTCTTTGCCTGAGTATTTG
Kdr Forward Primer	AATGGTACAGAAATGGAAGG
Kdr Reverse Primer	GCATCTCTTTCAGTCACTTC
Cdh5 Forward Primer	GGTTGTCATTCTCATCCAAG
Cdh5 Reverse Primer	AAAGAACTGGACAGAGAAAC
Actb Forward Primer	GATGTATGAAGGCTTTGGTC
Actb Reverse Primer	TGTGCACTTTTATTGGTCTC
Tgfb1 Forward Primer	GGATACCAACTATTGCTTCAG
Tgfb1 Reverse Primer	TGTCCAGGCTCCAAATATAG
Smad2 Forward Primer	TAATCACAGCTTGGATTTGC
Smad2 Reverse Primer	CTGGTTTAGTTCATAGTATGCG
Smad3 Forward Primer	CAATATTCCAGAAACCCCAC
Smad3 Reverse Primer	CTGTAGGTCCAAGTTATTGTG
ACTA2 Forward Primer	AGATCAAGATCATTGCCCC
ACTA2 Reverse Primer	TTCATCGTATTCCTGTTTGC
COL1A1 Forward Primer	GCTATGATGAGAAATCAACCG
COL1A1 Reverse Primer	TCATCTCCATTCTTTCCAGG
FN1 Forward Primer	CCATAGCTGAGAAGTGTTTTG
FN1 Reverse Primer	CAAGTACAATCTACCATCATCC
S100A4 Forward Primer	AAGTTCAAGCTCAACAAGTC
S100A4 Reverse Primer	CAGCTTCATCTGTCCTTTTC
KDR Forward Primer	GTACATAGTTGTCGTTGTAGG

KDR Reverse Primer	TCAATCCCCACATTTAGTTC
PECAM1 Forward Primer	AGATACTCTAGAACGGAAGG
PECAM1 Reverse Primer	CAGAGGTCTTGAAATACAGG
CDH5 Forward Primer	CGCAATAGACAAGGACATAAC
CDH5 Reverse Primer	TATCGTGATTATCCGTGAGG
ACTB Forward Primer	GACGACATGGAGAAAATCTG
ACTB Reverse Primer	ATGATCTGGGTCATCTTCTC
TGFB1 Forward Primer	AACCCACAACGAAATCTATG
TGFB1 Reverse Primer	CTTTTAACTTGAGCCTCAGC
SMAD2 Forward Primer	CAGTTTTGCCTCCAGTATTAG
SMAD2 Reverse Primer	AGTGAGTATAGTCATCCAGAG
